# Measurement of top quark–antiquark pair production in association with a W or Z boson in pp collisions at $$\sqrt{s} = 8$$$$\,\text {TeV}$$

**DOI:** 10.1140/epjc/s10052-014-3060-7

**Published:** 2014-09-17

**Authors:** The CMS Collaboration, V. Khachatryan, A. M. Sirunyan, A. Tumasyan, W. Adam, T. Bergauer, M. Dragicevic, J. Erö, C. Fabjan, M. Friedl, R. Frühwirth, V. M. Ghete, C. Hartl, N. Hörmann, J. Hrubec, M. Jeitler, W. Kiesenhofer, V. Knünz, M. Krammer, I. Krätschmer, D. Liko, I. Mikulec, D. Rabady, B. Rahbaran, H. Rohringer, R. Schöfbeck, J. Strauss, A. Taurok, W. Treberer-Treberspurg, W. Waltenberger, C.-E. Wulz, V. Mossolov, N. Shumeiko, J. Suarez Gonzalez, S. Alderweireldt, M. Bansal, S. Bansal, T. Cornelis, E. A. De Wolf, X. Janssen, A. Knutsson, S. Luyckx, S. Ochesanu, B. Roland, R. Rougny, M. Van De Klundert, H. Van Haevermaet, P. Van Mechelen, N. Van Remortel, A. Van Spilbeeck, F. Blekman, S. Blyweert, J. D’Hondt, N. Daci, N. Heracleous, J. Keaveney, S. Lowette, M. Maes, A. Olbrechts, Q. Python, D. Strom, S. Tavernier, W. Van Doninck, P. Van Mulders, G. P. Van Onsem, I. Villella, C. Caillol, B. Clerbaux, G. De Lentdecker, D. Dobur, L. Favart, A. P. R. Gay, A. Grebenyuk, A. Léonard, A. Mohammadi, L. Perniè, T. Reis, T. Seva, L. Thomas, C. Vander Velde, P. Vanlaer, J. Wang, V. Adler, K. Beernaert, L. Benucci, A. Cimmino, S. Costantini, S. Crucy, S. Dildick, A. Fagot, G. Garcia, J. Mccartin, A. A. Ocampo Rios, D. Ryckbosch, S. Salva Diblen, M. Sigamani, N. Strobbe, F. Thyssen, M. Tytgat, E. Yazgan, N. Zaganidis, S. Basegmez, C. Beluffi, G. Bruno, R. Castello, A. Caudron, L. Ceard, G. G. Da Silveira, C. Delaere, T. du Pree, D. Favart, L. Forthomme, A. Giammanco, J. Hollar, P. Jez, M. Komm, V. Lemaitre, C. Nuttens, D. Pagano, L. Perrini, A. Pin, K. Piotrzkowski, A. Popov, L. Quertenmont, M. Selvaggi, M. Vidal Marono, J. M. Vizan Garcia, N. Beliy, T. Caebergs, E. Daubie, G. H. Hammad, W. L. Aldá Júnior, G. A. Alves, L. Brito, M. Correa Martins Junior, T. Dos Reis Martins, C. Mora Herrera, M. E. Pol, W. Carvalho, J. Chinellato, A. Custódio, E. M. Da Costa, D. De Jesus Damiao, C. De Oliveira Martins, S. Fonseca De Souza, H. Malbouisson, D. Matos Figueiredo, L. Mundim, H. Nogima, W. L. Prado Da Silva, J. Santaolalla, A. Santoro, A. Sznajder, E. J. Tonelli Manganote, A. Vilela Pereira, C. A. Bernardes, S. Dogra, T. R. Fernandez Perez Tomei, E. M. Gregores, P. G. Mercadante, S. F. Novaes, Sandra S. Padula, A. Aleksandrov, V. Genchev, P. Iaydjiev, A. Marinov, S. Piperov, M. Rodozov, S. Stoykova, G. Sultanov, V. Tcholakov, M. Vutova, A. Dimitrov, I. Glushkov, R. Hadjiiska, V. Kozhuharov, L. Litov, B. Pavlov, P. Petkov, J. G. Bian, G. M. Chen, H. S. Chen, M. Chen, R. Du, C. H. Jiang, S. Liang, R. Plestina, J. Tao, X. Wang, Z. Wang, C. Asawatangtrakuldee, Y. Ban, Y. Guo, Q. Li, W. Li, S. Liu, Y. Mao, S. J. Qian, D. Wang, L. Zhang, W. Zou, C. Avila, L. F. Chaparro Sierra, C. Florez, J. P. Gomez, B. Gomez Moreno, J. C. Sanabria, N. Godinovic, D. Lelas, D. Polic, I. Puljak, Z. Antunovic, M. Kovac, V. Brigljevic, K. Kadija, J. Luetic, D. Mekterovic, L. Sudic, A. Attikis, G. Mavromanolakis, J. Mousa, C. Nicolaou, F. Ptochos, P. A. Razis, M. Bodlak, M. Finger, M. Finger, Y. Assran, A. Ellithi Kamel, M. A. Mahmoud, A. Radi, M. Kadastik, M. Murumaa, M. Raidal, A. Tiko, P. Eerola, G. Fedi, M. Voutilainen, J. Härkönen, V. Karimäki, R. Kinnunen, M. J. Kortelainen, T. Lampén, K. Lassila-Perini, S. Lehti, T. Lindén, P. Luukka, T. Mäenpää, T. Peltola, E. Tuominen, J. Tuominiemi, E. Tuovinen, L. Wendland, T. Tuuva, M. Besancon, F. Couderc, M. Dejardin, D. Denegri, B. Fabbro, J. L. Faure, C. Favaro, F. Ferri, S. Ganjour, A. Givernaud, P. Gras, G. Hamel de Monchenault, P. Jarry, E. Locci, J. Malcles, J. Rander, A. Rosowsky, M. Titov, S. Baffioni, F. Beaudette, P. Busson, C. Charlot, T. Dahms, M. Dalchenko, L. Dobrzynski, N. Filipovic, A. Florent, R. Granier de Cassagnac, L. Mastrolorenzo, P. Miné, C. Mironov, I. N. Naranjo, M. Nguyen, C. Ochando, P. Paganini, S. Regnard, R. Salerno, J. B. Sauvan, Y. Sirois, C. Veelken, Y. Yilmaz, A. Zabi, J.-L. Agram, J. Andrea, A. Aubin, D. Bloch, J.-M. Brom, E. C. Chabert, C. Collard, E. Conte, J.-C. Fontaine, D. Gelé, U. Goerlach, C. Goetzmann, A.-C. Le Bihan, P. Van Hove, S. Gadrat, S. Beauceron, N. Beaupere, G. Boudoul, E Bouvier, S. Brochet, C. A. Carrillo Montoya, J. Chasserat, R. Chierici, D. Contardo, P. Depasse, H. El Mamouni, J. Fan, J. Fay, S. Gascon, M. Gouzevitch, B. Ille, T. Kurca, M. Lethuillier, L. Mirabito, S. Perries, J. D. Ruiz Alvarez, D. Sabes, L. Sgandurra, V. Sordini, M. Vander Donckt, P. Verdier, S. Viret, H. Xiao, Z. Tsamalaidze, C. Autermann, S. Beranek, M. Bontenackels, M. Edelhoff, L. Feld, O. Hindrichs, K. Klein, A. Ostapchuk, A. Perieanu, F. Raupach, J. Sammet, S. Schael, H. Weber, B. Wittmer, V. Zhukov, M. Ata, E. Dietz-Laursonn, D. Duchardt, M. Erdmann, R. Fischer, A. Güth, T. Hebbeker, C. Heidemann, K. Hoepfner, D. Klingebiel, S. Knutzen, P. Kreuzer, M. Merschmeyer, A. Meyer, P. Millet, M. Olschewski, K. Padeken, P. Papacz, H. Reithler, S. A. Schmitz, L. Sonnenschein, D. Teyssier, S. Thüer, M. Weber, V. Cherepanov, Y. Erdogan, G. Flügge, H. Geenen, M. Geisler, W. Haj Ahmad, A. Heister, F. Hoehle, B. Kargoll, T. Kress, Y. Kuessel, J. Lingemann, A. Nowack, I. M. Nugent, L. Perchalla, O. Pooth, A. Stahl, I. Asin, N. Bartosik, J. Behr, W. Behrenhoff, U. Behrens, A. J. Bell, M. Bergholz, A. Bethani, K. Borras, A. Burgmeier, A. Cakir, L. Calligaris, A. Campbell, S. Choudhury, F. Costanza, C. Diez Pardos, S. Dooling, T. Dorland, G. Eckerlin, D. Eckstein, T. Eichhorn, G. Flucke, J. Garay Garcia, A. Geiser, P. Gunnellini, J. Hauk, G. Hellwig, M. Hempel, D. Horton, H. Jung, A. Kalogeropoulos, M. Kasemann, P. Katsas, J. Kieseler, C. Kleinwort, D. Krücker, W. Lange, J. Leonard, K. Lipka, A. Lobanov, W. Lohmann, B. Lutz, R. Mankel, I. Marfin, I.-A. Melzer-Pellmann, A. B. Meyer, J. Mnich, A. Mussgiller, S. Naumann-Emme, A. Nayak, O. Novgorodova, F. Nowak, E. Ntomari, H. Perrey, D. Pitzl, R. Placakyte, A. Raspereza, P. M. Ribeiro Cipriano, E. Ron, M. Ö. Sahin, J. Salfeld-Nebgen, P. Saxena, R. Schmidt, T. Schoerner-Sadenius, M. Schröder, C. Seitz, S. Spannagel, A. D. R. Vargas Trevino, R. Walsh, C. Wissing, M. Aldaya Martin, V. Blobel, M. Centis Vignali, A. r. Draeger, J. Erfle, E. Garutti, K. Goebel, M. Görner, J. Haller, M. Hoffmann, R. S. Höing, H. Kirschenmann, R. Klanner, R. Kogler, J. Lange, T. Lapsien, T. Lenz, I. Marchesini, J. Ott, T. Peiffer, N. Pietsch, J. Poehlsen, T. Poehlsen, D. Rathjens, C. Sander, H. Schettler, P. Schleper, E. Schlieckau, A. Schmidt, M. Seidel, V. Sola, H. Stadie, G. Steinbrück, D. Troendle, E. Usai, L. Vanelderen, C. Barth, C. Baus, J. Berger, C. Böser, E. Butz, T. Chwalek, W. De Boer, A. Descroix, A. Dierlamm, M. Feindt, F. Frensch, M. Giffels, F. Hartmann, T. Hauth, U. Husemann, I. Katkov, A. Kornmayer, E. Kuznetsova, P. Lobelle Pardo, M. U. Mozer, Th. Müller, A. Nürnberg, G. Quast, K. Rabbertz, F. Ratnikov, S. Röcker, H. J. Simonis, F. M. Stober, R. Ulrich, J. Wagner-Kuhr, S. Wayand, T. Weiler, R. Wolf, G. Anagnostou, G. Daskalakis, T. Geralis, V. A. Giakoumopoulou, A. Kyriakis, D. Loukas, A. Markou, C. Markou, A. Psallidas, I. Topsis-Giotis, A. Panagiotou, N. Saoulidou, E. Stiliaris, X. Aslanoglou, I. Evangelou, G. Flouris, C. Foudas, P. Kokkas, N. Manthos, I. Papadopoulos, E. Paradas, G. Bencze, C. Hajdu, P. Hidas, D. Horvath, F. Sikler, V. Veszpremi, G. Vesztergombi, A. J. Zsigmond, N. Beni, S. Czellar, J. Karancsi, J. Molnar, J. Palinkas, Z. Szillasi, P. Raics, Z. L. Trocsanyi, B. Ujvari, S. K. Swain, S. B. Beri, V. Bhatnagar, N. Dhingra, R. Gupta, U. Bhawandeep, A. K. Kalsi, M. Kaur, M. Mittal, N. Nishu, J. B. Singh, Ashok Kumar, Arun Kumar, S. Ahuja, A. Bhardwaj, B. C. Choudhary, A. Kumar, S. Malhotra, M. Naimuddin, K. Ranjan, V. Sharma, S. Banerjee, S. Bhattacharya, K. Chatterjee, S. Dutta, B. Gomber, Sa. Jain, Sh. Jain, R. Khurana, A. Modak, S. Mukherjee, D. Roy, S. Sarkar, M. Sharan, A. Abdulsalam, D. Dutta, S. Kailas, V. Kumar, A. K. Mohanty, L. M. Pant, P. Shukla, A. Topkar, T. Aziz, S. Banerjee, S. Bhowmik, R. M. Chatterjee, R. K. Dewanjee, S. Dugad, S. Ganguly, S. Ghosh, M. Guchait, A. Gurtu, G. Kole, S. Kumar, M. Maity, G. Majumder, K. Mazumdar, G. B. Mohanty, B. Parida, K. Sudhakar, N. Wickramage, H. Bakhshiansohi, H. Behnamian, S. M. Etesami, A. Fahim, R. Goldouzian, A. Jafari, M. Khakzad, M. Mohammadi Najafabadi, M. Naseri, S. Paktinat Mehdiabadi, B. Safarzadeh, M. Zeinali, M. Felcini, M. Grunewald, M. Abbrescia, L. Barbone, C. Calabria, S. S. Chhibra, A. Colaleo, D. Creanza, N. De Filippis, M. De Palma, L. Fiore, G. Iaselli, G. Maggi, M. Maggi, S. My, S. Nuzzo, A. Pompili, G. Pugliese, R. Radogna, G. Selvaggi, L. Silvestris, G. Singh, R. Venditti, P. Verwilligen, G. Zito, G. Abbiendi, A. C. Benvenuti, D. Bonacorsi, S. Braibant-Giacomelli, L. Brigliadori, R. Campanini, P. Capiluppi, A. Castro, F. R. Cavallo, G. Codispoti, M. Cuffiani, G. M. Dallavalle, F. Fabbri, A. Fanfani, D. Fasanella, P. Giacomelli, C. Grandi, L. Guiducci, S. Marcellini, G. Masetti, A. Montanari, F. L. Navarria, A. Perrotta, F. Primavera, A. M. Rossi, T. Rovelli, G. P. Siroli, N. Tosi, R. Travaglini, S. Albergo, G. Cappello, M. Chiorboli, S. Costa, F. Giordano, R. Potenza, A. Tricomi, C. Tuve, G. Barbagli, V. Ciulli, C. Civinini, R. D’Alessandro, E. Focardi, E. Gallo, S. Gonzi, V. Gori, P. Lenzi, M. Meschini, S. Paoletti, G. Sguazzoni, A. Tropiano, L. Benussi, S. Bianco, F. Fabbri, D. Piccolo, F. Ferro, M. Lo Vetere, E. Robutti, S. Tosi, M. E. Dinardo, S. Fiorendi, S. Gennai, R. Gerosa, A. Ghezzi, P. Govoni, M. T. Lucchini, S. Malvezzi, R. A. Manzoni, A. Martelli, B. Marzocchi, D. Menasce, L. Moroni, M. Paganoni, D. Pedrini, S. Ragazzi, N. Redaelli, T. Tabarelli de Fatis, S. Buontempo, N. Cavallo, S. Di Guida, F. Fabozzi, A. O. M. Iorio, L. Lista, S. Meola, M. Merola, P. Paolucci, P. Azzi, N. Bacchetta, D. Bisello, A. Branca, R. Carlin, P. Checchia, M. Dall’Osso, T. Dorigo, U. Dosselli, F. Fanzago, M. Galanti, F. Gasparini, U. Giubilato, A. Gozzelino, K. Kanishchev, S. Lacaprara, M. Margoni, A. T. Meneguzzo, F. Montecassiano, J. Pazzini, N. Pozzobon, P. Ronchese, F. Simonetto, E. Torassa, M. Tosi, P. Zotto, A. Zucchetta, G. Zumerle, M. Gabusi, S. P. Ratti, C. Riccardi, P. Salvini, P. Vitulo, M. Biasini, G. M. Bilei, D. Ciangottini, L. Fanò, P. Lariccia, G. Mantovani, M. Menichelli, F. Romeo, A. Saha, A. Santocchia, A. Spiezia, K. Androsov, P. Azzurri, G. Bagliesi, J. Bernardini, T. Boccali, G. Broccolo, R. Castaldi, M. A. Ciocci, R. Dell’Orso, S. Donato, F. Fiori, L. Foà, A. Giassi, M. T. Grippo, F. Ligabue, T. Lomtadze, L. Martini, A. Messineo, C. S. Moon, F. Palla, A. Rizzi, A. Savoy-Navarro, A. T. Serban, P. Spagnolo, P. Squillacioti, R. Tenchini, G. Tonelli, A. Venturi, P. G. Verdini, C. Vernieri, L. Barone, F. Cavallari, G. D’imperio, D. Del Re, M. Diemoz, M. Grassi, C. Jorda, E. Longo, F. Margaroli, P. Meridiani, F. Micheli, S. Nourbakhsh, G. Organtini, R. Paramatti, S. Rahatlou, C. Rovelli, F. Santanastasio, L. Soffi, P. Traczyk, N. Amapane, R. Arcidiacono, S. Argiro, M. Arneodo, R. Bellan, C. Biino, N. Cartiglia, S. Casasso, M. Costa, A. Degano, N. Demaria, L. Finco, C. Mariotti, S. Maselli, E. Migliore, V. Monaco, M. Musich, M. M. Obertino, G. Ortona, L. Pacher, N. Pastrone, M. Pelliccioni, G. L. Pinna Angioni, A. Potenza, A. Romero, M. Ruspa, R. Sacchi, A. Solano, A. Staiano, U. Tamponi, S. Belforte, V. Candelise, M. Casarsa, F. Cossutti, G. Della Ricca, B. Gobbo, C. La Licata, M. Marone, D. Montanino, A. Schizzi, T. Umer, A. Zanetti, T. J. Kim, S. Chang, A. Kropivnitskaya, S. K. Nam, D. H. Kim, G. N. Kim, M. S. Kim, M. S. Kim, D. J. Kong, S. Lee, Y. D. Oh, H. Park, A. Sakharov, D. C. Son, J. Y. Kim, S. Song, S. Choi, D. Gyun, B. Hong, M. Jo, H. Kim, Y. Kim, B. Lee, K. S. Lee, S. K. Park, Y. Roh, M. Choi, J. H. Kim, I. C. Park, S. Park, G. Ryu, M. S. Ryu, Y. Choi, Y. K. Choi, J. Goh, D. Kim, E. Kwon, J. Lee, H. Seo, I. Yu, A. Juodagalvis, J. R. Komaragiri, M. A. B. Md Ali, H. Castilla-Valdez, E. De La Cruz-Burelo, I. Heredia-de La Cruz, R. Lopez-Fernandez, A. Sanchez-Hernandez, S. Carrillo Moreno, F. Vazquez Valencia, I. Pedraza, H. A. Salazar Ibarguen, E. Casimiro Linares, A. Morelos Pineda, D. Krofcheck, P. H. Butler, S. Reucroft, A. Ahmad, M. Ahmad, Q. Hassan, H. R. Hoorani, S. Khalid, W. A. Khan, T. Khurshid, M. A. Shah, M. Shoaib, H. Bialkowska, M. Bluj, B. Boimska, T. Frueboes, M. Górski, M. Kazana, K. Nawrocki, K. Romanowska-Rybinska, M. Szleper, P. Zalewski, G. Brona, K. Bunkowski, M. Cwiok, W. Dominik, K. Doroba, A. Kalinowski, M. Konecki, J. Krolikowski, M. Misiura, M. Olszewski, W. Wolszczak, P. Bargassa, C. Beirão Da Cruz E Silva, P. Faccioli, P. G. Ferreira Parracho, M. Gallinaro, F. Nguyen, J. Rodrigues Antunes, J. Seixas, J. Varela, P. Vischia, I. Golutvin, V. Karjavin, V. Konoplyanikov, V. Korenkov, G. Kozlov, A. Lanev, A. Malakhov, V. Matveev, V.V Mitsyn, P. Moisenz, V. Palichik, V. Perelygin, S. Shmatov, S. Shulha, N. Skatchkov, V. Smirnov, E. Tikhonenko, A. Zarubin, V. Golovtsov, Y. Ivanov, V. Kim, P. Levchenko, V. Murzin, V. Oreshkin, I. Smirnov, V. Sulimov, L. Uvarov, S. Vavilov, A. Vorobyev, An. Vorobyev, Yu. Andreev, A. Dermenev, S. Gninenko, N. Golubev, M. Kirsanov, N. Krasnikov, A. Pashenkov, D. Tlisov, A. Toropin, V. Epshteyn, V. Gavrilov, N. Lychkovskaya, V. Popov, G. Safronov, S. Semenov, A. Spiridonov, V. Stolin, E. Vlasov, A. Zhokin, V. Andreev, M. Azarkin, I. Dremin, M. Kirakosyan, A. Leonidov, G. Mesyats, S. V. Rusakov, A. Vinogradov, A. Belyaev, E. Boos, V. Bunichev, M. Dubinin, L. Dudko, A. Ershov, A. Gribushin, V. Klyukhin, I. Lokhtin, S. Obraztsov, M. Perfilov, V. Savrin, A. Snigirev, I. Azhgirey, I. Bayshev, S. Bitioukov, V. Kachanov, A. Kalinin, D. Konstantinov, V. Krychkine, V. Petrov, R. Ryutin, A. Sobol, L. Tourtchanovitch, S. Troshin, N. Tyurin, A. Uzunian, A. Volkov, P. Adzic, M. Ekmedzic, J. Milosevic, V. Rekovic, J. Alcaraz Maestre, C. Battilana, E. Calvo, M. Cerrada, M. Chamizo Llatas, N. Colino, B. De La Cruz, A. Delgado Peris, D. Domínguez Vázquez, A. Escalante Del Valle, C. Fernandez Bedoya, J. P. Fernández Ramos, J. Flix, M. C. Fouz, P. Garcia-Abia, O. Gonzalez Lopez, S. Goy Lopez, J. M. Hernandez, M. I. Josa, G. Merino, E. Navarro De Martino, A. Pérez-Calero Yzquierdo, J. Puerta Pelayo, A. Quintario Olmeda, I. Redondo, L. Romero, M. S. Soares, C. Albajar, J. F. de Trocóniz, M. Missiroli, D. Moran, H. Brun, J. Cuevas, J. Fernandez Menendez, S. Folgueras, I. Gonzalez Caballero, L. Lloret Iglesias, J. A. Brochero Cifuentes, I. J. Cabrillo, A. Calderon, J. Duarte Campderros, M. Fernandez, G. Gomez, A. Graziano, A. Lopez Virto, J. Marco, R. Marco, C. Martinez Rivero, F. Matorras, F. J. Munoz Sanchez, J. Piedra Gomez, T. Rodrigo, A. Y. Rodríguez-Marrero, A. Ruiz-Jimeno, L. Scodellaro, I. Vila, R. Vilar Cortabitarte, D. Abbaneo, E. Auffray, G. Auzinger, M. Bachtis, P. Baillon, A. H. Ball, D. Barney, A. Benaglia, J. Bendavid, L. Benhabib, J. F. Benitez, C. Bernet, G. Bianchi, P. Bloch, A. Bocci, A. Bonato, O. Bondu, C. Botta, H. Breuker, T. Camporesi, G. Cerminara, S. Colafranceschi, M. D’Alfonso, D. d’Enterria, A. Dabrowski, A. David, F. De Guio, A. De Roeck, S. De Visscher, M. Dobson, M. Dordevic, N. Dupont-Sagorin, A. Elliott-Peisert, J. Eugster, G. Franzoni, W. Funk, D. Gigi, K. Gill, D. Giordano, M. Girone, F. Glege, R. Guida, S. Gundacker, M. Guthoff, R. Guida, J. Hammer, M. Hansen, P. Harris, J. Hegeman, V. Innocente, P. Janot, K. Kousouris, K. Krajczar, P. Lecoq, C. Lourenço, N. Magini, L. Malgeri, M. Mannelli, J. Marrouche, L. Masetti, F. Meijers, S. Mersi, E. Meschi, F. Moortgat, S. Morovic, M. Mulders, P. Musella, L. Orsini, L. Pape, E. Perez, L. Perrozzi, A. Petrilli, G. Petrucciani, A. Pfeiffer, M. Pierini, M. Pimiä, D. Piparo, M. Plagge, A. Racz, G. Rolandi, M. Rovere, H. Sakulin, C. Schäfer, C. Schwick, A. Sharma, P. Siegrist, P. Silva, M. Simon, P. Sphicas, D. Spiga, J. Steggemann, B. Stieger, M. Stoye, D. Treille, A. Tsirou, G. I. Veres, J. R. Vlimant, N. Wardle, H. K. Wöhri, H. Wollny, W. D. Zeuner, W. Bertl, K. Deiters, W. Erdmann, R. Horisberger, Q. Ingram, H. C. Kaestli, D. Kotlinski, U. Langenegger, D. Renker, T. Rohe, F. Bachmair, L. Bäni, L. Bianchini, P. Bortignon, M. A. Buchmann, B. Casal, N. Chanon, A. Deisher, G. Dissertori, M. Dittmar, M. Donegà, M. Dünser, P. Eller, C. Grab, D. Hits, W. Lustermann, B. Mangano, A. C. Marini, P. Martinez Ruiz del Arbol, D. Meister, N. Mohr, C. Nägeli, F. Nessi-Tedaldi, F. Pandolfi, F. Pauss, M. Peruzzi, M. Quittnat, L. Rebane, M. Rossini, A. Starodumov, M. Takahashi, K. Theofilatos, R. Wallny, H. A. Weber, C. Amsler, M. F. Canelli, V. Chiochia, A. De Cosa, A. Hinzmann, T. Hreus, B. Kilminster, C. Lange, B. Millan Mejias, J. Ngadiuba, P. Robmann, F. J. Ronga, S. Taroni, M. Verzetti, Y. Yang, M. Cardaci, K. H. Chen, C. Ferro, C. M. Kuo, W. Lin, Y. J. Lu, R. Volpe, S. S. Yu, P. Chang, Y. H. Chang, Y. W. Chang, Y. Chao, K. F. Chen, P. H. Chen, C. Dietz, U. Grundler, W.-S. Hou, K. Y. Kao, Y. J. Lei, Y. F. Liu, R.-S. Lu, D. Majumder, E. Petrakou, Y. M. Tzeng, R. Wilken, B. Asavapibhop, N. Srimanobhas, N. Suwonjandee, A. Adiguzel, M. N. Bakirci, S. Cerci, C. Dozen, I. Dumanoglu, E. Eskut, S. Girgis, G. Gokbulut, E. Gurpinar, I. Hos, E. E. Kangal, A. Kayis Topaksu, G. Onengut, K. Ozdemir, S. Ozturk, A. Polatoz, K. Sogut, D. Sunar Cerci, B. Tali, H. Topakli, M. Vergili, I. V. Akin, B. Bilin, S. Bilmis, H. Gamsizkan, G. Karapinar, K. Ocalan, S. Sekmen, U. E. Surat, M. Yalvac, M. Zeyrek, E. Gülmez, B. Isildak, M. Kaya, O. Kaya, H. Bahtiyar, E. Barlas, K. Cankocak, F. I. Vardarlı, M. Yücel, L. Levchuk, P. Sorokin, J. J. Brooke, E. Clement, D. Cussans, H. Flacher, R. Frazier, J. Goldstein, M. Grimes, G. P. Heath, H. F. Heath, J. Jacob, L. Kreczko, C. Lucas, Z. Meng, D. M. Newbold, S. Paramesvaran, A. Poll, S. Senkin, V. J. Smith, T. Williams, K. W. Bell, A. Belyaev, C. Brew, R. M. Brown, D. J. A. Cockerill, J. A. Coughlan, K. Harder, S. Harper, E. Olaiya, D. Petyt, C. H. Shepherd-Themistocleous, A. Thea, I. R. Tomalin, W. J. Womersley, S. D. Worm, M. Baber, R. Bainbridge, O. Buchmuller, D. Burton, D. Colling, N. Cripps, M. Cutajar, P. Dauncey, G. Davies, M. Della Negra, P. Dunne, W. Ferguson, J. Fulcher, D. Futyan, A. Gilbert, G. Hall, G. Iles, M. Jarvis, G. Karapostoli, M. Kenzie, R. Lane, R. Lucas, L. Lyons, A.-M. Magnan, S. Malik, B. Mathias, J. Nash, A. Nikitenko, J. Pela, M. Pesaresi, K. Petridis, D. M. Raymond, S. Rogerson, A. Rose, C. Seez, P. Sharp, A. Tapper, M. Vazquez Acosta, T. Virdee, J. E. Cole, P. R. Hobson, A. Khan, P. Kyberd, D. Leggat, D. Leslie, W. Martin, I. D. Reid, P. Symonds, L. Teodorescu, M. Turner, J. Dittmann, K. Hatakeyama, A. Kasmi, H. Liu, T. Scarborough, O. Charaf, S. I. Cooper, C. Henderson, P. Rumerio, A. Avetisyan, T. Bose, C. Fantasia, P. Lawson, C. Richardson, J. Rohlf, D. Sperka, J. St. John, L. Sulak, J. Alimena, E. Berry, S. Bhattacharya, G. Christopher, D. Cutts, Z. Demiragli, A. Ferapontov, A. Garabedian, U. Heintz, G. Kukartsev, E. Laird, G. Landsberg, M. Luk, M. Narain, M. Segala, T. Sinthuprasith, T. Speer, J. Swanson, R. Breedon, G. Breto, M. Calderon De La Barca Sanchez, S. Chauhan, M. Chertok, J. Conway, R. Conway, P. T. Cox, R. Erbacher, M. Gardner, W. Ko, R. Lander, T. Miceli, M. Mulhearn, D. Pellett, J. Pilot, F. Ricci-Tam, M. Searle, S. Shalhout, J. Smith, M. Squires, D. Stolp, M. Tripathi, S. Wilbur, R. Yohay, R. Cousins, P. Everaerts, C. Farrell, J. Hauser, M. Ignatenko, G. Rakness, E. Takasugi, V. Valuev, M. Weber, J. Babb, K. Burt, R. Clare, J. Ellison, J. W. Gary, G. Hanson, J. Heilman, M. Ivova Rikova, P. Jandir, E. Kennedy, F. Lacroix, H. Liu, O. R. Long, A. Luthra, M. Malberti, H. Nguyen, M. Olmedo Negrete, A. Shrinivas, S. Sumowidagdo, S. Wimpenny, W. Andrews, J. G. Branson, G. B. Cerati, S. Cittolin, R. T. D’Agnolo, D. Evans, A. Holzner, R. Kelley, D. Klein, M. Lebourgeois, J. Letts, I. Macneill, D. Olivito, S. Padhi, C. Palmer, M. Pieri, M. Sani, V. Sharma, S. Simon, E. Sudano, M. Tadel, Y. Tu, A. Vartak, C. Welke, F. Würthwein, A. Yagil, J. Yoo, D. Barge, J. Bradmiller-Feld, C. Campagnari, T. Danielson, A. Dishaw, K. Flowers, M. Franco Sevilla, P. Geffert, C. George, F. Golf, L. Gouskos, J. Incandela, C. Justus, N. Mccoll, J. Richman, D. Stuart, W. To, C. West, A. Apresyan, A. Bornheim, J. Bunn, Y. Chen, E. Di Marco, J. Duarte, A. Mott, H. B. Newman, C. Pena, C. Rogan, M. Spiropulu, V. Timciuc, R. Wilkinson, S. Xie, R. Y. Zhu, V. Azzolini, A. Calamba, B. Carlson, T. Ferguson, Y. Iiyama, M. Paulini, J. Russ, H. Vogel, I. Vorobiev, J. P. Cumalat, W. T. Ford, A. Gaz, E. Luiggi Lopez, U. Nauenberg, J. G. Smith, K. Stenson, K. A. Ulmer, S. R. Wagner, J. Alexander, A. Chatterjee, J. Chu, S. Dittmer, N. Eggert, N. Mirman, G. Nicolas Kaufman, J. R. Patterson, A. Ryd, E. Salvati, L. Skinnari, W. Sun, W. D. Teo, J. Thom, J. Thompson, J. Tucker, Y. Weng, L. Winstrom, P. Wittich, D. Winn, S. Abdullin, M. Albrow, J. Anderson, G. Apollinari, L. A. T. Bauerdick, A. Beretvas, J. Berryhill, P. C. Bhat, K. Burkett, J. N. Butler, H. W. K. Cheung, F. Chlebana, S. Cihangir, V. D. Elvira, I. Fisk, J. Freeman, Y. Gao, E. Gottschalk, L. Gray, D. Green, S. Grünendahl, O. Gutsche, J. Hanlon, D. Hare, R. M. Harris, J. Hirschauer, B. Hooberman, S. Jindariani, M. Johnson, U. Joshi, K. Kaadze, B. Klima, B. Kreis, S. Kwan, J. Linacre, D. Lincoln, R. Lipton, T. Liu, J. Lykken, K. Maeshima, J. M. Marraffino, V. I. Martinez Outschoorn, S. Maruyama, D. Mason, P. McBride, K. Mishra, S. Mrenna, Y. Musienko, S. Nahn, C. Newman-Holmes, V. O’Dell, O. Prokofyev, E. Sexton-Kennedy, S. Sharma, A. Soha, W. J. Spalding, L. Spiegel, L. Taylor, S. Tkaczyk, N. V. Tran, L. Uplegger, E. W. Vaandering, R. Vidal, A. Whitbeck, J. Whitmore, F. Yang, D. Acosta, P. Avery, D. Bourilkov, M. Carver, T. Cheng, D. Curry, S. Das, M. De Gruttola, G. P. Di Giovanni, R. D. Field, M. Fisher, I. K. Furic, J. Hugon, J. Konigsberg, A. Korytov, T. Kypreos, J. F. Low, K. Matchev, P. Milenovic, G. Mitselmakher, L. Muniz, A. Rinkevicius, L. Shchutska, M. Snowball, J. Yelton, M. Zakaria, S. Hewamanage, S. Linn, P. Markowitz, G. Martinez, J. L. Rodriguez, T. Adams, A. Askew, J. Bochenek, B. Diamond, J. Haas, S. Hagopian, V. Hagopian, K. F. Johnson, H. Prosper, V. Veeraraghavan, M. Weinberg, M. M. Baarmand, M. Hohlmann, H. Kalakhety, F. Yumiceva, M. R. Adams, L. Apanasevich, V. E. Bazterra, D. Berry, R. R. Betts, I. Bucinskaite, R. Cavanaugh, O. Evdokimov, L. Gauthier, C. E. Gerber, D. J. Hofman, S. Khalatyan, P. Kurt, D. H. Moon, C. O’Brien, C. Silkworth, P. Turner, N. Varelas, E. A. Albayrak, B. Bilki, W. Clarida, K. Dilsiz, F. Duru, M. Haytmyradov, J.-P. Merlo, H. Mermerkaya, A. Mestvirishvili, A. Moeller, J. Nachtman, H. Ogul, Y. Onel, F. Ozok, A. Penzo, R. Rahmat, S. Sen, P. Tan, E. Tiras, J. Wetzel, T. Yetkin, K. Yi, B. A. Barnett, B. Blumenfeld, S. Bolognesi, D. Fehling, A. V. Gritsan, P. Maksimovic, C. Martin, M. Swartz, P. Baringer, A. Bean, G. Benelli, C. Bruner, J. Gray, R. P. Kenny, M. Malek, M. Murray, D. Noonan, S. Sanders, J. Sekaric, R. Stringer, Q. Wang, J. S. Wood, A. F. Barfuss, I. Chakaberia, A. Ivanov, S. Khalil, M. Makouski, Y. Maravin, L. K. Saini, S. Shrestha, N. Skhirtladze, I. Svintradze, J. Gronberg, D. Lange, F. Rebassoo, D. Wright, A. Baden, A. Belloni, B. Calvert, S. C. Eno, J. A. Gomez, N. J. Hadley, R. G. Kellogg, T. Kolberg, Y. Lu, M. Marionneau, A. C. Mignerey, K. Pedro, A. Skuja, M. B. Tonjes, S. C. Tonwar, A. Apyan, R. Barbieri, G. Bauer, W. Busza, I. A. Cali, M. Chan, L. Di Matteo, V. Dutta, G. Gomez Ceballos, M. Goncharov, D. Gulhan, M. Klute, Y. S. Lai, Y.-J. Lee, A. Levin, P. D. Luckey, T. Ma, C. Paus, D. Ralph, C. Roland, G. Roland, G. S. F. Stephans, F. Stöckli, K. Sumorok, D. Velicanu, J. Veverka, B. Wyslouch, M. Yang, A. S. Yoon, M. Zanetti, V. Zhukova, B. Dahmes, A. De Benedetti, A. Gude, S. C. Kao, K. Klapoetke, Y. Kubota, J. Mans, N. Pastika, R. Rusack, A. Singovsky, N. Tambe, J. Turkewitz, J. G. Acosta, L. M. Cremaldi, R. Kroeger, S. Oliveros, L. Perera, D. A. Sanders, D. Summers, E. Avdeeva, K. Bloom, S. Bose, D. R. Claes, A. Dominguez, R. Gonzalez Suarez, J. Keller, D. Knowlton, I. Kravchenko, J. Lazo-Flores, S. Malik, F. Meier, G. R. Snow, J. Dolen, A. Godshalk, I. Iashvili, S. Jain, A. Kharchilava, A. Kumar, S. Rappoccio, G. Alverson, E. Barberis, D. Baumgartel, M. Chasco, J. Haley, A. Massironi, D. Nash, T. Orimoto, D. Trocino, D. Wood, J. Zhang, A. Anastassov, K. A. Hahn, A. Kubik, L. Lusito, N. Mucia, N. Odell, B. Pollack, A. Pozdnyakov, M. Schmitt, S. Stoynev, K. Sung, M. Velasco, S. Won, A. Brinkerhoff, K. M. Chan, A. Drozdetskiy, M. Hildreth, C. Jessop, D. J. Karmgard, N. Kellams, K. Lannon, W. Luo, S. Lynch, N. Marinelli, T. Pearson, M. Planer, R. Ruchti, N. Valls, M. Wayne, M. Wolf, A. Woodard, L. Antonelli, J. Brinson, B. Bylsma, L. S. Durkin, S. Flowers, C. Hill, R. Hughes, K. Kotov, T. Y. Ling, D. Puigh, M. Rodenburg, G. Smith, B. L. Winer, H. Wolfe, H. W. Wulsin, O. Driga, P. Elmer, P. Hebda, A. Hunt, S. A. Koay, P. Lujan, D. Marlow, T. Medvedeva, M. Mooney, J. Olsen, P. Piroué, X. Quan, H. Saka, D. Stickland, C. Tully, J. S. Werner, S. C. Zenz, A. Zuranski, E. Brownson, H. Mendez, J. E. Ramirez Vargas, E. Alagoz, V. E. Barnes, D. Benedetti, G. Bolla, D. Bortoletto, M. De Mattia, Z. Hu, M. K. Jha, M. Jones, K. Jung, M. Kress, N. Leonardo, D. Lopes Pegna, V. Maroussov, P. Merkel, D. H. Miller, N. Neumeister, B. C. Radburn-Smith, X. Shi, I. Shipsey, D. Silvers, A. Svyatkovskiy, F. Wang, W. Xie, L. Xu, H. D. Yoo, J. Zablocki, Y. Zheng, N. Parashar, J. Stupak, A. Adair, B. Akgun, K. M. Ecklund, F. J. M. Geurts, W. Li, B. Michlin, B. P. Padley, R. Redjimi, J. Roberts, J. Zabel, B. Betchart, A. Bodek, R. Covarelli, P. de Barbaro, R. Demina, Y. Eshaq, T. Ferbel, A. Garcia-Bellido, P. Goldenzweig, J. Han, A. Harel, A. Khukhunaishvili, G. Petrillo, D. Vishnevskiy, R. Ciesielski, L. Demortier, K. Goulianos, G. Lungu, C. Mesropian, S. Arora, A. Barker, J. P. Chou, C. Contreras-Campana, E. Contreras-Campana, D. Duggan, D. Ferencek, Y. Gershtein, R. Gray, E. Halkiadakis, D. Hidas, A. Lath, S. Panwalkar, M. Park, R. Patel, S. Salur, S. Schnetzer, S. Somalwar, R. Stone, S. Thomas, P. Thomassen, M. Walker, K. Rose, S. Spanier, A. York, O. Bouhali, R. Eusebi, W. Flanagan, J. Gilmore, T. Kamon, V. Khotilovich, V. Krutelyov, R. Montalvo, I. Osipenkov, Y. Pakhotin, A. Perloff, J. Roe, A. Rose, A. Safonov, T. Sakuma, I. Suarez, A. Tatarinov, N. Akchurin, C. Cowden, J. Damgov, C. Dragoiu, P. R. Dudero, J. Faulkner, K. Kovitanggoon, S. Kunori, S. W. Lee, T. Libeiro, I. Volobouev, E. Appelt, A. G. Delannoy, S. Greene, A. Gurrola, W. Johns, C. Maguire, Y. Mao, A. Melo, M. Sharma, P. Sheldon, B. Snook, S. Tuo, J. Velkovska, M. W. Arenton, S. Boutle, B. Cox, B. Francis, J. Goodell, R. Hirosky, A. Ledovskoy, H. Li, C. Lin, C. Neu, J. Wood, C. Clarke, R. Harr, P. E. Karchin, C. Kottachchi Kankanamge Don, P. Lamichhane, J. Sturdy, D. A. Belknap, D. Carlsmith, M. Cepeda, S. Dasu, L. Dodd, S. Duric, E. Friis, R. Hall-Wilton, M. Herndon, A. Hervé, P. Klabbers, A. Lanaro, C. Lazaridis, A. Levine, R. Loveless, A. Mohapatra, I. Ojalvo, T. Perry, G. A. Pierro, G. Polese, I. Ross, T. Sarangi, A. Savin, W. H. Smith, C. Vuosalo, N. Woods, [Authorinst]The CMS Collaboration

**Affiliations:** 1 Yerevan Physics Institute, Yerevan, Armenia; 2 Institut für Hochenergiephysik der OeAW, Wien, Austria; 3 National Centre for Particle and High Energy Physics, Minsk, Belarus; 4 Universiteit Antwerpen, Antwerp, Belgium; 5 Vrije Universiteit Brussel, Brussel, Belgium; 6 Université Libre de Bruxelles, Bruxelles, Belgium; 7 Ghent University, Ghent, Belgium; 8 Université Catholique de Louvain, Louvain-la-Neuve, Belgium; 9 Université de Mons, Mons, Belgium; 10 Centro Brasileiro de Pesquisas Fisicas, Rio de Janeiro, Brazil; 11 Universidade do Estado do Rio de Janeiro, Rio de Janeiro, Brazil; 12 Universidade Estadual Paulista, Universidade Federal do ABC, São Paulo, Brazil; 13 Institute for Nuclear Research and Nuclear Energy, Sofia, Bulgaria; 14 University of Sofia, Sofia, Bulgaria; 15 Institute of High Energy Physics, Beijing, China; 16 State Key Laboratory of Nuclear Physics and Technology, Peking University, Beijing, China; 17 Universidad de Los Andes, Bogotá, Colombia; 18 Technical University of Split, Split, Croatia; 19 University of Split, Split, Croatia; 20 Institute Rudjer Boskovic, Zagreb, Croatia; 21 University of Cyprus, Nicosia, Cyprus; 22 Charles University, Prague, Czech Republic; 23 Academy of Scientific Research and Technology of the Arab Republic of Egypt, Egyptian Network of High Energy Physics, Cairo, Egypt; 24 National Institute of Chemical Physics and Biophysics, Tallinn, Estonia; 25 Department of Physics, University of Helsinki, Helsinki, Finland; 26 Helsinki Institute of Physics, Helsinki, Finland; 27 Lappeenranta University of Technology, Lappeenranta, Finland; 28 DSM/IRFU, CEA/Saclay, Gif-sur-Yvette, France; 29 Laboratoire Leprince-Ringuet, Ecole Polytechnique, IN2P3-CNRS, Palaiseau, France; 30 Institut Pluridisciplinaire Hubert Curien, Université de Strasbourg, Université de Haute Alsace Mulhouse, CNRS/IN2P3, Strasbourg, France; 31 Centre de Calcul de l’Institut National de Physique Nucleaire et de Physique des Particules, CNRS/IN2P3, Villeurbanne, France; 32 Institut de Physique Nucléaire de Lyon, Université de Lyon, Université Claude Bernard Lyon 1, CNRS-IN2P3, Villeurbanne, France; 33 Institute of High Energy Physics and Informatization, Tbilisi State University, Tbilisi, Georgia; 34 RWTH Aachen University, I. Physikalisches Institut, Aachen, Germany; 35 RWTH Aachen University, III. Physikalisches Institut A, Aachen, Germany; 36 RWTH Aachen University, III. Physikalisches Institut B, Aachen, Germany; 37 Deutsches Elektronen-Synchrotron, Hamburg, Germany; 38 University of Hamburg, Hamburg, Germany; 39 Institut für Experimentelle Kernphysik, Karlsruhe, Germany; 40 Institute of Nuclear and Particle Physics (INPP), NCSR Demokritos, Aghia Paraskevi, Greece; 41 University of Athens, Athens, Greece; 42 University of Ioánnina, Ioannina, Greece; 43 Wigner Research Centre for Physics, Budapest, Hungary; 44 Institute of Nuclear Research ATOMKI, Debrecen, Hungary; 45 University of Debrecen, Debrecen, Hungary; 46 National Institute of Science Education and Research, Bhubaneswar, India; 47 Panjab University, Chandigarh, India; 48 University of Delhi, Delhi, India; 49 Saha Institute of Nuclear Physics, Kolkata, India; 50 Bhabha Atomic Research Centre, Mumbai, India; 51 Tata Institute of Fundamental Research, Mumbai, India; 52 Institute for Research in Fundamental Sciences (IPM), Tehran, Iran; 53 University College Dublin, Dublin, Ireland; 54 INFN Sezione di Bari, Università di Bari, Politecnico di Bari, Bari, Italy; 55 INFN Sezione di Bologna, Università di Bologna, Bologna, Italy; 56 INFN Sezione di Catania, Università di Catania, CSFNSM, Catania, Italy; 57 INFN Sezione di Firenze, Università di Firenze, Florence, Italy; 58 INFN Laboratori Nazionali di Frascati, Frascati, Italy; 59 INFN Sezione di Genova, Università di Genova, Genoa, Italy; 60 INFN Sezione di Milano-Bicocca, Università di Milano-Bicocca, Milan, Italy; 61 INFN Sezione di Napoli, Università di Napoli ’Federico II’, Università della Basilicata (Potenza), Università G. Marconi (Roma), Naples, Italy; 62 INFN Sezione di Padova, Università di Padova, Università di Trento (Trento), Padua, Italy; 63 INFN Sezione di Pavia, Università di Pavia, Pavia, Italy; 64 INFN Sezione di Perugia, Università di Perugia, Perugia, Italy; 65 INFN Sezione di Pisa, Università di Pisa, Scuola Normale Superiore di Pisa, Pisa, Italy; 66 INFN Sezione di Roma, Università di Roma, Rome, Italy; 67 INFN Sezione di Torino, Università di Torino, Università del Piemonte Orientale (Novara), Turin, Italy; 68 INFN Sezione di Trieste, Università di Trieste, Trieste, Italy; 69 Chonbuk National University, Chonju, Korea; 70 Kangwon National University, Chunchon, Korea; 71 Kyungpook National University, Taegu, Korea; 72 Chonnam National University, Institute for Universe and Elementary Particles, Kwangju, Korea; 73 Korea University, Seoul, Korea; 74 University of Seoul, Seoul, Korea; 75 Sungkyunkwan University, Suwon, Korea; 76 Vilnius University, Vilnius, Lithuania; 77 National Centre for Particle Physics, Universiti Malaya, Kuala Lumpur, Malaysia; 78 Centro de Investigacion y de Estudios Avanzados del IPN, Mexico City, Mexico; 79 Universidad Iberoamericana, Mexico City, Mexico; 80 Benemerita Universidad Autonoma de Puebla, Puebla, Mexico; 81 Universidad Autónoma de San Luis Potosí, San Luis Potosí, Mexico; 82 University of Auckland, Auckland, New Zealand; 83 University of Canterbury, Christchurch, New Zealand; 84 National Centre for Physics, Quaid-I-Azam University, Islamabad, Pakistan; 85 National Centre for Nuclear Research, Swierk, Poland; 86 Institute of Experimental Physics, Faculty of Physics, University of Warsaw, Warsaw, Poland; 87 Laboratório de Instrumentação e Física Experimental de Partículas, Lisbon, Portugal; 88 Joint Institute for Nuclear Research, Dubna, Russia; 89 Petersburg Nuclear Physics Institute, Gatchina (St. Petersburg), Russia; 90 Institute for Nuclear Research, Moscow, Russia; 91 Institute for Theoretical and Experimental Physics, Moscow, Russia; 92 P. N. Lebedev Physical Institute, Moscow, Russia; 93 Skobeltsyn Institute of Nuclear Physics, Lomonosov Moscow State University, Moscow, Russia; 94 State Research Center of Russian Federation, Institute for High Energy Physics, Protvino, Russia; 95 University of Belgrade, Faculty of Physics and Vinca Institute of Nuclear Sciences, Belgrade, Serbia; 96 Centro de Investigaciones Energéticas Medioambientales y Tecnológicas (CIEMAT), Madrid, Spain; 97 Universidad Autónoma de Madrid, Madrid, Spain; 98 Universidad de Oviedo, Oviedo, Spain; 99 Instituto de Física de Cantabria (IFCA), CSIC-Universidad de Cantabria, Santander, Spain; 100 CERN, European Organization for Nuclear Research, Geneva, Switzerland; 101 Paul Scherrer Institut, Villigen, Switzerland; 102 Institute for Particle Physics, ETH Zurich, Zurich, Switzerland; 103 Universität Zürich, Zurich, Switzerland; 104 National Central University, Chung-Li, Taiwan; 105 National Taiwan University (NTU), Taipei, Taiwan; 106 Chulalongkorn University, Faculty of Science, Department of Physics, Bangkok, Thailand; 107 Cukurova University, Adana, Turkey; 108 Physics Department, Middle East Technical University, Ankara, Turkey; 109 Bogazici University, Istanbul, Turkey; 110 Istanbul Technical University, Istanbul, Turkey; 111 National Scientific Center, Kharkov Institute of Physics and Technology, Kharkiv, Ukraine; 112 University of Bristol, Bristol, UK; 113 Rutherford Appleton Laboratory, Didcot, UK; 114 Imperial College, London, UK; 115 Brunel University, Uxbridge, UK; 116 Baylor University, Waco, USA; 117 The University of Alabama, Tuscaloosa, USA; 118 Boston University, Boston, USA; 119 Brown University, Providence, USA; 120 University of California, Davis, USA; 121 University of California, Los Angeles, USA; 122 University of California, Riverside, Riverside, USA; 123 University of California, San Diego, La Jolla, USA; 124 University of California, Santa Barbara, Santa Barbara, USA; 125 California Institute of Technology, Pasadena, USA; 126 Carnegie Mellon University, Pittsburgh, USA; 127 University of Colorado at Boulder, Boulder, USA; 128 Cornell University, Ithaca, USA; 129 Fairfield University, Fairfield, USA; 130 Fermi National Accelerator Laboratory, Batavia, USA; 131 University of Florida, Gainesville, USA; 132 Florida International University, Miami, USA; 133 Florida State University, Tallahassee, USA; 134 Florida Institute of Technology, Melbourne, USA; 135 University of Illinois at Chicago (UIC), Chicago, USA; 136 The University of Iowa, Iowa City, USA; 137 Johns Hopkins University, Baltimore, USA; 138 The University of Kansas, Lawrence, USA; 139 Kansas State University, Manhattan, USA; 140 Lawrence Livermore National Laboratory, Livermore, USA; 141 University of Maryland, College Park, USA; 142 Massachusetts Institute of Technology, Cambridge, USA; 143 University of Minnesota, Minneapolis, USA; 144 University of Mississippi, Oxford, USA; 145 University of Nebraska-Lincoln, Lincoln, USA; 146 State University of New York at Buffalo, Buffalo, USA; 147 Northeastern University, Boston, USA; 148 Northwestern University, Evanston, USA; 149 University of Notre Dame, Notre Dame, USA; 150 The Ohio State University, Columbus, USA; 151 Princeton University, Princeton, USA; 152 University of Puerto Rico, Mayagüez, USA; 153 Purdue University, West Lafayette, USA; 154 Purdue University Calumet, Hammond, USA; 155 Rice University, Houston, USA; 156 University of Rochester, Rochester, USA; 157 The Rockefeller University, New York, USA; 158 Rutgers, The State University of New Jersey, Piscataway, USA; 159 University of Tennessee, Knoxville, USA; 160 Texas A&M University, College Station, USA; 161 Texas Tech University, Lubbock, USA; 162 Vanderbilt University, Nashville, USA; 163 University of Virginia, Charlottesville, USA; 164 Wayne State University, Detroit, USA; 165 University of Wisconsin, Madison, USA; 166 CERN, Geneva, Switzerland; 167 CERN, 1211 Geneva 23, Switzerland

## Abstract

A measurement of the cross section for the production of top quark–antiquark pairs (${\mathrm {t}}\overline{{\mathrm {t}}}$) in association with a vector boson V (W or Z) in proton-proton collisions at $\sqrt{s} = 8$ $\,\text {TeV}$ is presented. The results are based on a dataset corresponding to an integrated luminosity of 19.5 fb$^{-1}$ recorded with the CMS detector at the LHC. The measurement is performed in three leptonic (e and $\upmu$) channels: a same-sign dilepton analysis targeting ${\mathrm {t}}\overline{{\mathrm {t}}} \mathrm {W}$ events, and trilepton and four-lepton analyses designed for ${\mathrm {t}}\overline{{\mathrm {t}}} {\mathrm {Z}}$ events. In the same-sign dilepton channel, the ${\mathrm {t}}\overline{{\mathrm {t}}} \mathrm {W}$ cross section is measured as $\sigma _{{\mathrm {t}}\overline{{\mathrm {t}}} \mathrm {W}} = 170 ^{+90}_{-80}\,\text {(stat)} \pm 70\,\text {(syst)} \, \text {fb}$, corresponding to a significance of 1.6 standard deviations over the background-only hypothesis. Combining the trilepton and four-lepton channels, a direct measurement of the ${\mathrm {t}}\overline{{\mathrm {t}}} {\mathrm {Z}}$ cross section, $\sigma _{{\mathrm {t}}\overline{{\mathrm {t}}} {\mathrm {Z}}} = 200 ^{+80}_{-70}\,\text {(stat)} ^{+40}_{-30}\,\text {(syst)} \mathrm{fb}^{-1}$, is obtained with a significance of 3.1 standard deviations. The measured cross sections are compatible with standard model predictions within their experimental uncertainties. The inclusive ${\mathrm {t}}\overline{{\mathrm {t}}} {\mathrm {V}}$ process is observed with a significance of 3.7 standard deviations from the combination of all three leptonic channels.

## Introduction

Two decades after the discovery of the top quark [1, 2], many of its properties are still to be determined or are only loosely constrained by experimental data. Among these properties are the couplings between the top quark and the vector bosons.

The existence of non-zero couplings between the top quark and the neutral vector bosons can be inferred through the analysis of direct production of ${\mathrm {t}}\overline{{\mathrm {t}}}$ pairs in association with a $\gamma$ or a ${\mathrm {Z}}$ boson. The CERN LHC allows these two processes to be disentangled and the corresponding couplings to be measured. The associated production of ${\mathrm {t}}\overline{{\mathrm {t}}}$ pairs with a W boson, the ${\mathrm {t}}\overline{{\mathrm {t}}} \mathrm {W}$ process, has a cross section similar to ${\mathrm {t}}\overline{{\mathrm {t}}} {\mathrm {Z}}$ and ${\mathrm {t}}\overline{{\mathrm {t}}} \gamma$ production. All three processes can be used to test the internal consistency of the standard model (SM) [3–5] and search for the presence of new physics. Despite their small cross sections, they are significant backgrounds to analyses that probe phenomena with even smaller, or comparable, cross sections. Examples are searches for supersymmetry [6–8] in same-sign dilepton [9] and in multilepton [10] final states, and the analysis of the SM ${\mathrm {t}}\overline{{\mathrm {t}}} {\mathrm {H}}$ process with the Higgs boson and the top quarks decaying leptonically.

The measurement of the ${\mathrm {t}}\overline{{\mathrm {t}}} \gamma$ process has been documented by the CDF Collaboration [11] for proton-antiproton collisions at a centre-of-mass energy $\sqrt{s}=1.96\,\text {TeV}$. This article presents instead the measurement of cross sections for the ${\mathrm {t}}\overline{{\mathrm {t}}} \mathrm {W}$ and ${\mathrm {t}}\overline{{\mathrm {t}}} {\mathrm {Z}}$ processes in proton-proton (pp) collisions at $\sqrt{s}=8\,\text {TeV}$. The analysis is based on data corresponding to an integrated luminosity of 19.5$\,\text {fb}^-1$ collected with the CMS detector at the LHC in 2012. Unlike the previous observation of the ${\mathrm {t}}\overline{{\mathrm {t}}} {\mathrm {V}}$ process (${\mathrm {V}}$ equal to $\mathrm {W}$ or ${\mathrm {Z}}$) at $\sqrt{s}=7\,\text {TeV}$ [12], here the ${\mathrm {t}}\overline{{\mathrm {t}}} \mathrm {W}$ process is treated separately.

Three leptonic ($\mathrm {e}$ and ${\upmu }$) final states are considered: same-sign dilepton events, trilepton events, and four-lepton events. The same-sign dilepton events are used for the measurement of the ${\mathrm {t}}\overline{{\mathrm {t}}} \mathrm {W}$ process, where one lepton originates from the leptonic decay of one of the two top quarks and the other like-sign lepton is produced in the decay of the prompt vector boson. The trilepton events are used for the identification of ${\mathrm {t}}\overline{{\mathrm {t}}} {\mathrm {Z}}$ events in which one lepton is again produced from the leptonic decay of one of the two top quarks, and the two other opposite-sign and same-flavour leptons stem from the decay of the Z boson. The four-lepton events are used to identify ${\mathrm {t}}\overline{{\mathrm {t}}} {\mathrm {Z}}$ events in which both the top quarks and the Z boson decay leptonically. For all three signatures, signal events containing leptonic $\tau$ decays are implicitly included.

**Fig. 1:**
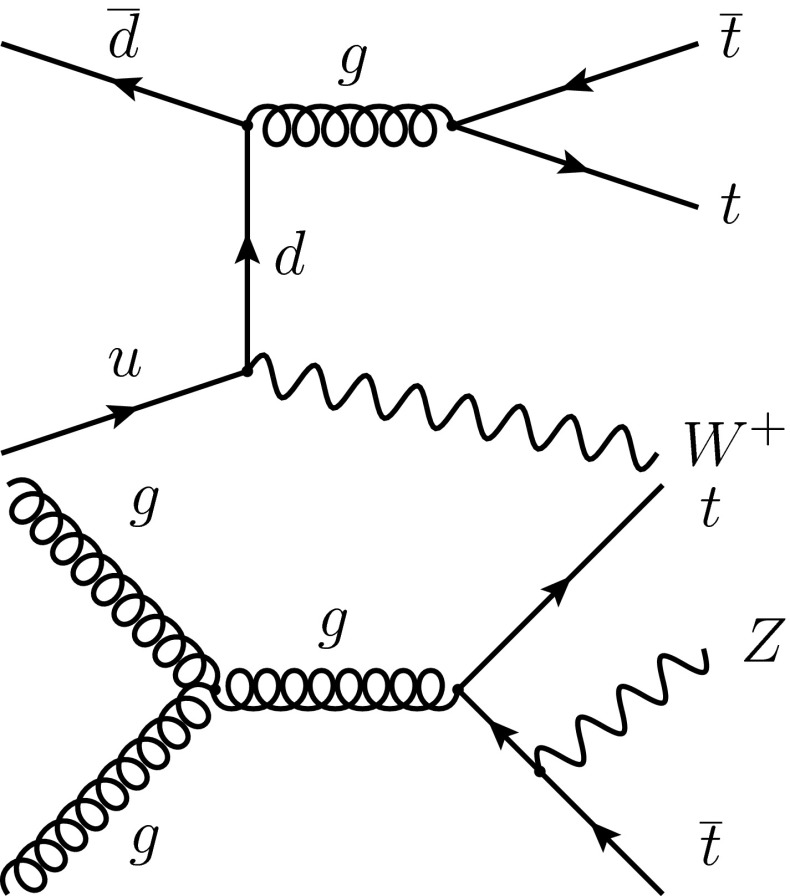
The dominant leading-order Feynman diagrams for ${\mathrm {t}}\overline{{\mathrm {t}}} \mathrm {W}$ and ${\mathrm {t}}\overline{{\mathrm {t}}} {\mathrm {Z}}$ production in pp collisions. The charge conjugate of the diagrams shown is implied

Figure 1 shows the most important leading-order Feynman diagrams for ${\mathrm {t}}\overline{{\mathrm {t}}} \mathrm {W}$ and ${\mathrm {t}}\overline{{\mathrm {t}}} {\mathrm {Z}}$ production in pp collisions. For pp collisions at $\sqrt{s}=8\,\text {TeV}$, the current best estimates of the cross sections for these processes are based on quantum chromodynamics (QCD) calculations at next-to-leading-order (NLO) in $\alpha _s$. Using CT10 NLO [13] parton distribution functions (PDF) and a top-quark mass of 173$\,\text {GeV}$, the software framework MadGraph 5_amc@nlo  [14, 15] provides a cross section of $206^{+21}_{-23}\, \text {fb}$ for ${\mathrm {t}}\overline{{\mathrm {t}}} \mathrm {W}$ production and of $197^{+22}_{-25}\, \text {fb}$ for ${\mathrm {t}}\overline{{\mathrm {t}}} {\mathrm {Z}}$ production, in agreement with independent NLO calculations [16, 17].

As the number of selected signal events is expected to be comparable to that of the background processes, the estimation of the background is a key aspect of the analysis. The strategy is to use background-dominated control samples in data to the maximum extent possible. Many contributions to the background, in particular those caused by detector misreconstruction, are estimated in this way, while the remaining irreducible backgrounds are estimated using Monte Carlo (MC) simulations and the most precise calculations of cross sections that are available. For the three separate channels and also for their combination, the yields of events found in excess of the expected backgrounds are used to measure the corresponding signal cross sections.

## The CMS detector

The central feature of the CMS apparatus is a superconducting solenoid of 6$\text {\,m}$ internal diameter, providing a magnetic field of 3.8$\text {\,T}$. Within the superconducting solenoid volume are a silicon pixel and strip tracker, a lead tungstate crystal electromagnetic calorimeter, and a brass/scintillator hadron calorimeter. Muons are measured in gas-ionization detectors embedded in the steel flux-return yoke outside the solenoid. A forward calorimeter extends the coverage provided by the barrel and endcap detectors. CMS uses a right-handed coordinate system, with the origin at the nominal interaction point, the $x$ axis pointing to the centre of the LHC, the $y$ axis pointing up (perpendicular to the LHC plane), and the $z$ axis along the anticlockwise-beam direction. The polar angle $\theta$ is measured from the positive $z$ axis and the azimuthal angle $\phi$ is measured in the $x$-$y$ plane in radians. Events are selected by a two-stage trigger system: a hardware-based trigger followed by a software-based high-level trigger running on the data acquisition computer farm. A more detailed description of the CMS apparatus can be found in Ref. [18].

## Event selection and Monte Carlo simulation

For all the channels considered in this analysis, the data are selected online by dilepton ($\mathrm {e}\mathrm {e}$, $\mathrm {e}{\upmu }$, and ${\upmu }{\upmu }$) triggers that demand a transverse momentum ($p_{\mathrm {T}}$) larger than 17$\,\text {GeV}$ for the highest $p_{\mathrm {T}}$ lepton and 8$\,\text {GeV}$ for the second-highest. The online selection involves loose identification for both flavours and isolation requirements on electrons. Other channel-specific triggers, which are described in detail later, select control regions that are used for the estimation of specific backgrounds and the assessment of the signal selection efficiency. After the online selection, data and simulated events are reconstructed offline using the same software.

Each event is processed using a global event reconstruction approach  [19, 20]. This consists in reconstructing and identifying particles using an optimized combination of the information from all subdetectors. In this process, the identification of the particle type (photon, electron, muon, charged hadron, and neutral hadron) plays an important role in the determination of the particle direction and energy.

The tracks reconstructed in the silicon tracker are clustered in several primary vertices corresponding to the different pp interactions occurring within the same LHC bunch crossing. The vertex that has the largest $\sum _i p_{\mathrm {T}_i}^{2}$, where $i$ runs over all tracks of the vertex, is assumed to identify the signal primary vertex. Its position is used to discriminate against particles originating from the other interactions (pileup) and to distinguish between prompt and non-prompt particles stemming from the signal interaction.

For each event, hadronic jets are clustered from the reconstructed particles using the anti-$k_{\mathrm {T}}$ algorithm [21], operated with a distance parameter of 0.5. The jet momentum is determined as the vector sum of all particle momenta in the jet. In this analysis the jets used for the definition of the signal regions (signal jets) are required to be inside the tracker acceptance, i.e. $|\eta |<2.4$ where $\eta \equiv -\ln [ \tan (\theta /2)]$, to reduce the uncertainty in the jet reconstruction efficiency and improve the precision of the energy measurement. Jet energy corrections are applied to account for the non-linear response of the calorimeters and other instrumental effects. These corrections are based on in situ measurements using dijet and $\gamma +\text {jet}$ data samples [22]. A two-fold approach is employed to reduce the effect of pileup during jet reconstruction. Firstly, charged particles whose trajectories point to pileup vertices are excluded from the set of particles that are used for the reconstruction of signal jets. Secondly, the average energy density due to neutral pileup particles is evaluated in each event, and the corresponding energy inside the jet is subtracted [23]. Then a jet identification requirement [24], primarily based on the energy balance between charged and neutral hadrons in a jet, is applied to remove jets that are misreconstructed or originate from instrumental noise. Finally, the trajectories of all the charged particles of a jet are used to calculate a $p_{\mathrm {T}}$-averaged longitudinal impact parameter for each signal jet [25]. This variable is then employed as a discriminator against jets from pileup. Unless otherwise specified, signal jets are required to have $p_{\mathrm {T}} > 30$ $\,\text {GeV}$.

To identify (tag) jets originating from the hadronization of bottom quarks (b jets), the combined secondary vertex (CSV) algorithm [26] is used. The algorithm combines the information about track impact parameters and secondary vertices within jets in a likelihood discriminant to provide separation between b jets and jets originating from light quarks, gluons, or charm quarks. We use here two operating points. The *loose* working point corresponds to a b-tagging efficiency for jets originating from ${\mathrm {b}}$ quarks of about 85 % and a misidentification probability for jets from light quarks and gluons of 10 %. The *medium* working point provides an efficiency of about 70 % and a misidentification probability of 1.5 %.

Muons and electrons are identified using standard quality criteria [27, 28] and are required to have $p_{\mathrm {T}} > 20$ $\,\text {GeV}$ and $|\eta |<2.4$. For the four-lepton channel only, identified leptons with $p_{\mathrm {T}}$ between 10 and 20$\,\text {GeV}$ are also employed for the event selection. To reduce the contamination caused by leptons from heavy-flavour decays or misidentified hadrons in jets, leptons are required to be isolated and to pass a selection on the impact parameter, which is calculated with respect to the position of the signal primary vertex. Candidates are considered isolated when the ratio of the scalar sum of the transverse momenta of all the other reconstructed particles in a cone of $\Delta R = \sqrt{{(\Delta \eta )^2 + (\Delta \phi )^2}} = 0.3$ around the candidate, relative to the lepton $p_{\mathrm {T}}$ value, is less than 5–10 %, the exact value of the threshold depending on the flavour of the lepton and on the final state. This relative isolation is corrected for the expected contribution from pileup using an approach that is similar to the one employed for the reconstruction of jets [29]. The leptons are required to originate from the primary interaction demanding that their transverse and longitudinal impact parameters are smaller than 50–200$\,\mu \text {m}$ and 0.1–1.0$\,\text {cm}$, respectively. The tightest selections in these ranges are used for the lepton flavour and final states that are most affected by backgrounds due to non-prompt leptons.

Finally, the observables $E_{\mathrm {T}}^{\text {miss}}$ and $H_{\mathrm {T}}$ are used, respectively, to identify the presence of neutrinos and to measure the hadronic activity in the analysed events. The former is defined as the magnitude of the vector sum of the transverse momenta of all reconstructed particles, the latter is the scalar sum of the transverse momenta of all signal jets.

Simulations, which include pileup effects, are used to estimate some of the backgrounds, as well as to calculate the selection efficiency for the ${\mathrm {t}}\overline{{\mathrm {t}}} \mathrm {W}$ and ${\mathrm {t}}\overline{{\mathrm {t}}} {\mathrm {Z}}$ signal events. Simulated samples are generated with the MadGraph 5 [30] program, with the exception of the ${\mathrm {t}}\overline{{\mathrm {t}}} {\mathrm {H}}$ background process that is generated using pythia  6 [31]. All simulated samples are processed using a Geant4-based model [32] of the CMS detector. Signal samples are produced with MadGraph 5, which is used with the CTEQ6L1 [33] PDF and is interfaced to pythia  6.424 to simulate parton showering and hadronization.

## Same-sign dilepton analysis

The aim of the same-sign dilepton analysis is to search for ${\mathrm {t}}\overline{{\mathrm {t}}} \mathrm {W}$ events where one lepton is produced in the leptonic decay chain of one of the two top quarks, and the other like-sign lepton stems directly from the decay of the prompt vector boson:

$$\begin{aligned} \mathrm {p}\mathrm {p}\rightarrow {\mathrm {t}}\overline{{\mathrm {t}}} \mathrm {W} \rightarrow ({\mathrm {t}}\rightarrow {\mathrm {b}}\ell \nu )({\mathrm {t}}\rightarrow {\mathrm {b}}{\mathrm {q}}\overline{{\mathrm {q}}}')(\mathrm {W} \rightarrow \ell \nu ), \end{aligned}$$

where $\ell$ corresponds to an electron or a muon. By requiring that the two selected leptons have the same sign, only half of the signal produced in the dilepton final state can be selected. However, the requirement significantly improves the signal-over-background ratio. The main background is caused by misidentification and misreconstruction effects: decay products of heavy-flavour mesons that give rise to non-prompt leptons and pions in jets misidentified as prompt leptons. A second, smaller, source of background is also caused by misreconstruction and consists of opposite-sign dilepton events where the charge of one of the two leptons is wrongly assigned.

The selection for the dilepton channel is conducted through the following steps:

1. Each event must contain two isolated leptons of the same charge and $p_{\mathrm {T}} > 40\,\text {GeV}$. Both leptons are required to be compatible with the signal primary vertex and have a relative isolation smaller than $5\,\%$. The invariant mass of the dilepton pair is required to be larger than 8$\,\text {GeV}$.
2. Three or more signal jets must be reconstructed, and at least one of these has to be b-tagged using the medium working point of the CSV algorithm.
3. Events are rejected if they contain a third lepton forming, with one of the other two leptons, a same-flavour opposite-sign pair whose invariant mass is within 15$\,\text {GeV}$ of the known Z-boson mass [34]. For the third lepton, the relative isolation must be less than $9\,(10)$ % if it is an electron (muon), and the transverse momentum requirement is loosened to $p_{\mathrm {T}} > 10$ $\,\text {GeV}$.
4. The $H_{\mathrm {T}}$ value is required to be greater than 155$\,\text {GeV}$.
5. Selected events are grouped in three categories depending on the lepton flavour: $\mathrm {e}\mathrm {e}$, $\mathrm {e}{\upmu }$, and ${\upmu }{\upmu }$ dilepton pairs. Each of these categories is further split into two separate sets of dileptons with either positive or negative charges, for a total of six signal regions.

The tight-lepton selection (1) reduces the background from misidentified leptons, while the invariant mass requirement rejects events with pairs of energetic leptons from decays of heavy hadrons. The requirement (2) on the general number of jets and on the number of b-tagged jets present in the event decreases the background from electroweak processes, e.g. WZ production, that can have same-sign leptons in the final state, but are accompanied by little hadronic activity. The WZ background is also significantly reduced by the third-lepton veto (3). The $H_{\mathrm {T}}$ requirement (4) as well as the threshold on the lepton $p_{\mathrm {T}}$ (1) have been optimized for the best signal significance. This selection also minimizes the expected uncertainty in the measured cross section. The splitting (5) of the signal candidates in six categories is done for two reasons: exploiting the smaller background from lepton and charge misidentification in signal regions with muons and benefitting from the greater signal cross section in the plus-plus dilepton final states, which is caused by the abundance of quarks, instead of antiquarks, within the colliding protons at the LHC. Finally, the Z-boson veto is necessary to make the dilepton analysis statistically independent from the trilepton one described later. Events with three leptons are not rejected if they pass the Z-boson veto, since these can stem from a fully-leptonic decay of the ${\mathrm {t}}\overline{{\mathrm {t}}}$ pair in ${\mathrm {t}}\overline{{\mathrm {t}}} \mathrm {W}$ signal events.

### Background estimation

After the full same-sign dilepton selection is applied, there are three general categories of background processes that are selected together with ${\mathrm {t}}\overline{{\mathrm {t}}} \mathrm {W}$ signal events: background from non-prompt or misidentified leptons (*misidentified lepton* background); background from lepton charge misidentification (*mismeasured charge* background); WZ and ${\mathrm {t}}\overline{{\mathrm {t}}} {\mathrm {Z}}$ production, as well as other rare SM processes that contain genuine pairs of prompt, isolated and same-sign leptons. The subset of these processes that do not contain a Z boson in the final state forms the *irreducible* component of the background. This includes the production of like-sign WW and the production of the Higgs boson in association with a pair of top quarks. The production of a ${\mathrm {t}}\overline{{\mathrm {t}}}$ pair in association with a W boson by means of double parton scattering is expected to have a cross section two orders of magnitude smaller than the ${\mathrm {t}}\overline{{\mathrm {t}}} \mathrm {W}$ production through single scattering [35]. This source of background is therefore considered negligible and is ignored in the rest of the analysis.

The first background consists mostly of ${\mathrm {t}}\overline{{\mathrm {t}}}$ events, with a second important contribution coming from $\mathrm {W}\text {+jets}$ events. In both cases, one prompt lepton originates from the leptonic decay of a W boson, and another same-sign lepton is caused by the misidentification of a non-prompt lepton stemming from the decay of a heavy-flavour hadron. In $\mathrm {W}\text {+jets}$ events, smaller sources of misreconstructed leptons affecting this category of background are given by the misreconstruction of hadrons, the production of muons from light-meson decays, and the reconstruction of electrons from unidentified photon conversions. The background yield is estimated from data using a sample of events that satisfy the full analysis selection, except that one of the two leptons is required to pass a looser lepton selection and fail the full selection (sideband region). The background rate is then obtained weighting the events in this sideband region by the “tight-to-loose” ratio, i.e. the probability for a loosely identified lepton to pass the full set of requirements. This tight-to-loose ratio is measured as a function of lepton $p_{\mathrm {T}}$ and $\eta$ in a control sample of dijet events, which is depleted of prompt leptons and is selected by dedicated single-muon and single-electron triggers. The systematic uncertainty in the background estimate is due to the differences in the various sources of non-prompt or misidentified leptons, between the dijet events where the tight-to-loose ratio is measured and the sideband region where the ratio is applied. Among the most important differences are the $p_{\mathrm {T}}$ spectrum and the flavour of the jets containing the misidentified leptons. These two quantities have been varied in the control sample using appropriate selections and then the effects on the tight-to-loose ratio, and on the background estimate itself, have been quantified. The range of variation for these two quantities has been guided by a simulation of the background processes. The full systematic uncertainty in the background is estimated to be 50 %. The statistical part of the uncertainty is driven by the number of events in the sideband region and it is significantly smaller than the systematic uncertainty for all six signal regions.

The probability to misidentify the charge of muons is about an order of magnitude smaller than for electrons. Therefore the magnitude of the background caused by charge misidentification, mostly in Drell–Yan and ${\mathrm {t}}\overline{{\mathrm {t}}}$ events, is driven only by electrons. This background is estimated by selecting opposite-sign $\mathrm {e}$ $\mathrm {e}$ or $\mathrm {e}$ ${\upmu }$ events that pass the full analysis selection, except the same-sign requirement, and then weighting them by the $p_{\mathrm {T}}$- and $\eta$-dependent probability for electron charge misassignment. This probability and its variation as a function of the lepton $p_{\mathrm {T}}$ and $\eta$ are determined by combining information from simulation and a control data sample of ${\mathrm {Z}}\rightarrow \mathrm {e}\mathrm {e}$ events. For the electron selection used in this analysis, the probability of charge misidentification is about $10^{-4}$ and $10^{-3}$ for electrons reconstructed in the barrel and endcap detectors, respectively. The background estimate has an uncertainty of 30 % (15 %) for the $\mathrm {e}$ $\mathrm {e}$ ($\mathrm {e}$ ${\upmu }$) signal regions. This uncertainty accounts for differences between data and simulation, and the limited momentum range of electrons in the ${\mathrm {Z}}$-boson control sample.

Production of WZ and ${\mathrm {t}}\overline{{\mathrm {t}}} {\mathrm {Z}}$ events, and the irreducible backgrounds, are all estimated from simulation as done when calculating the signal selection efficiencies. For each SM process contributing to this category of background, the dominant systematic uncertainty is the one in the theoretical cross section prediction. Depending on the process, we use an uncertainty of 15–50 % and consider it as fully correlated across all signal regions.

### Same-sign dilepton results

After the full analysis selection is applied, 36 events are observed in data, to be compared with $25.2\pm 3.4\,(\text {syst}\oplus \text {stat})$ events expected from background processes and $39.7\pm 3.5\,(\text {syst}\oplus \text {stat})$ events from the sum of background and ${\mathrm {t}}\overline{{\mathrm {t}}} \mathrm {W}$ signal with the SM cross section. For both predictions, the statistical and systematic uncertainties are added in quadrature.

**Fig. 2:**
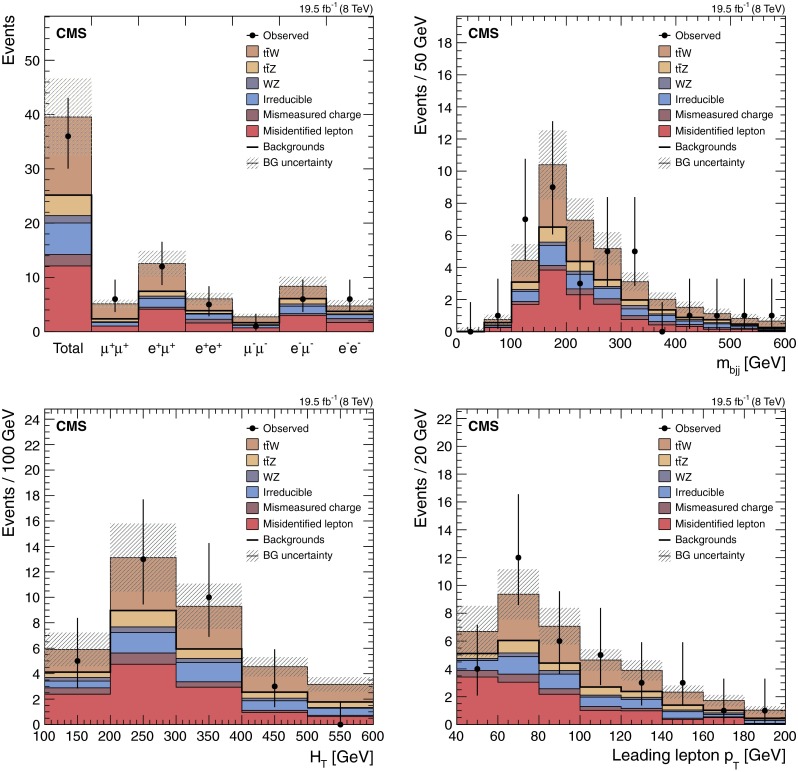
Event yields in data after final dilepton selection requirements, compared to the background estimates and signal expectations. Contributions separated by final states (*top left*), trijets mass distribution for the hadronic top-quark candidate (*top right*), $H_{\mathrm {T}}$ distribution (*bottom left*), and the leading-lepton $p_{\mathrm {T}}$ distribution (*bottom right*). The combination of statistical and systematic uncertainties is denoted by the *shaded area*

The event yields, along with the corresponding uncertainties for each background component, are reported in Table 1. The top left panel of Fig. 2 shows the distribution of the expected and observed events across the six different signal regions, and for all dilepton channels added together. As already anticipated, the positively charged channels are expected to collect a larger quantity of signal than the negatively charged channels, for a comparable quantity of background. The first three channels therefore drive the sensitivity of this analysis. In Table 1 and Fig. 2, and in the equivalent tables and figures for the other two leptonic channels, the uncertainty in the signal cross section is not shown because it does not affect the precision of the experimental measurement.

**Table 1:** Expected signal, estimated backgrounds, the sum of the two, and observed number of events for the $\upmu ^{\pm }\upmu ^{\pm }$, $\mathrm {e}^\pm \upmu ^{\pm }$, and $\mathrm {e}^\pm \mathrm {e}^\pm$ channels. Uncertainties include both the statistical and the systematic components. The systematic uncertainty in the signal contribution does not include the theoretical uncertainty in the signal production cross section

	$\mathrm {\upmu ^+}\mathrm {\upmu ^+}$	$\mathrm {e}^+\mathrm {\upmu ^+}$	$\mathrm {e}^+\mathrm {e}^+$	${\upmu ^-}{\upmu ^-}$	$\mathrm {e}^-{\upmu ^-}$	$\mathrm {e}^-\mathrm {e}^-$
${\mathrm {t}}\overline{{\mathrm {t}}} \mathrm {W}$ (expected)	$2.8 \pm 0.4$	$5.1 \pm 0.5$	$2.2 \pm 0.3$	$1.1 \pm 0.2$	$2.3 \pm 0.3$	$1.0 \pm 0.2$
Misidentified lepton	$1.0 \pm 0.6$	$4.1 \pm 2.1$	$1.6 \pm 0.9$	$0.7 \pm 0.4$	$3.0 \pm 1.5$	$1.7 \pm 0.9$
Mismeasured charge	–	$0.4 \pm 0.1$	$0.7 \pm 0.2$	–	$0.4 \pm 0.1$	$0.7 \pm 0.2$
Irreducible	$0.7 \pm 0.4$	$1.6 \pm 0.9$	$0.9 \pm 0.5$	$0.5 \pm 0.3$	$1.4 \pm 0.7$	$0.7 \pm 0.4$
WZ	$0.1 \pm 0.1$	$0.4 \pm 0.1$	$0.1 \pm 0.1$	$0.1 \pm 0.1$	$0.4 \pm 0.1$	$0.2 \pm 0.1$
${\mathrm {t}}\overline{{\mathrm {t}}} {\mathrm {Z}}$	$0.6 \pm 0.3$	$0.9 \pm 0.5$	$0.5 \pm 0.3$	$0.4 \pm 0.2$	$1.0 \pm 0.5$	$0.5 \pm 0.3$
Total background	$2.4 \pm 0.7$	$7.4 \pm 2.3$	$3.9 \pm 1.1$	$1.7 \pm 0.5$	$6.1 \pm 1.8$	$3.7 \pm 1.1$
Total expected	$5.2 \pm 0.8$	$12.5 \pm 2.4$	$6.1 \pm 1.1$	$2.8 \pm 0.5$	$8.4 \pm 1.8$	$4.7 \pm 1.1$
Observed	6	12	5	1	6	6

The other three panels of Fig. 2 show the distributions for the invariant mass $m_{\mathrm {bjj}}$ of the three jets expected to originate from the hadronic top-quark decay (top right), $H_{\mathrm {T}}$ (bottom left), and the leading-lepton $p_{\mathrm {T}}$ (bottom right) for all six signal regions combined together. For each event, the three signal jets used for the $m_{\mathrm {bjj}}$ distribution are selected as follows: one, and only one, of the three jets is b-tagged; among the possible three-jet combinations the one chosen minimizes $\Delta R_{\mathrm {jjj}} = \sqrt{{ (\Delta R_{\mathrm {j}_1,{\mathrm {t}}})^2 + (\Delta R_{\mathrm {j}_2,{\mathrm {t}}})^2 + (\Delta R_{\mathrm {j}_3,{\mathrm {t}}})^2 }}$, where $\Delta R_{\mathrm {j}_i,{\mathrm {t}}}$ is the $\Delta R$ distance between the direction of the $i$th jet and the direction of the reconstructed hadronic top-quark candidate. In all four distributions data and simulation are found in agreement. In particular, the $m_{\mathrm {bjj}}$ distribution confirms that most of the background from misidentified leptons is originating from top-quark events. In Fig. 2, and also in all other similar figures included in this document, the error bar enclosing each data point represents the 68 % confidence level interval around the mean of the corresponding Poisson distribution.

Based on the observed number of events, the background estimates, and the signal acceptance (including the leptonic branching fractions), the inclusive ${\mathrm {t}}\overline{{\mathrm {t}}} \mathrm {W}$ production cross section is measured, through the combination of the six dilepton channels, as

$$\begin{aligned} \sigma _{{\mathrm {t}}\overline{{\mathrm {t}}} \mathrm {W}} = 170 ^{+90}_{-80}\,\text {(stat)} \pm 70\,\text {(syst)} \, \text {fb}, \end{aligned}$$

including statistical and systematic uncertainties, compared to the SM expectation of $206^{+21}_{-23}\, \text {fb}$. The significance of the result over the background-only hypothesis is equivalent to 1.6 standard deviations (2.0 standard deviations expected).

The systematic uncertainty in the signal selection efficiency is 8 %. It is treated in a common way with the three- and four-lepton channels and is discussed in detail in Sect. 7. Additionally, for all channels there is a 2.6 % uncertainty in the expected yield of signal and simulation-derived background events because of the uncertainty in the luminosity normalization [36]. However, together with the low yield of signal events, the main factor dominating the uncertainty in the cross section measurement is the uncertainty in the largest background component, i.e. the 50 % uncertainty in the background from misidentified leptons.

## Trilepton analysis

The production of a ${\mathrm {t}}\overline{{\mathrm {t}}}$ pair in association with a ${\mathrm {Z}}$ boson is analysed in the final state with three high-energy, isolated, and prompt leptons. The trilepton analysis targets final states with only one $\mathrm {W}$ boson decaying leptonically:

$$\begin{aligned} \mathrm {p}\mathrm {p}\rightarrow {\mathrm {t}}\overline{{\mathrm {t}}} {\mathrm {Z}} \rightarrow ({\mathrm {t}}\rightarrow {\mathrm {b}}\ell \nu )({\mathrm {t}}\rightarrow {\mathrm {b}}{\mathrm {q}}\overline{{\mathrm {q}}}')({\mathrm {Z}}\rightarrow \ell \overline{\ell }). \end{aligned}$$

The event selection, described in more detail below, focuses on the main features of this final state: two oppositely charged leptons of the same flavour, consistent with the ${\mathrm {Z}}$-boson decay; an additional lepton; and at least four jets, at least two of which are b-tagged. The isolation of the leptons has additionally been loosened to reflect the diminished contribution of misidentified leptons to the background.

The selection for the trilepton channel is conducted through the following steps:

1. Each event must contain three isolated leptons of $p_{\mathrm {T}} > 20\,\text {GeV}$ and passing identification requirements described in Sect. 3. All three leptons are required to be compatible with the signal primary vertex and have a relative isolation smaller than 9 % (10 %) for electrons (muons).
2. Two of the leptons must be of the same flavour, be oppositely charged, and form an invariant mass between 81 and 101$\,\text {GeV}$ to be consistent with a ${\mathrm {Z}}$-boson decay. If multiple pairs pass this selection, the one with the mass closest to the known ${\mathrm {Z}}$-boson mass is selected as the ${\mathrm {Z}}$ boson candidate.
3. To match the final-state signal topology, four or more signal jets must be reconstructed with at least three of these jets having $p_{\mathrm {T}} >30\,\text {GeV}$, and the fourth jet is required to have $p_{\mathrm {T}} >15\,\text {GeV}$. Additional identification and pileup suppression selections are applied as described in Sect. 3.
4. At least two of the jets with $p_{\mathrm {T}} >30\,\text {GeV}$ must be b-tagged, the first using the medium working point of the CSV algorithm, and the second using the loose working point.
5. Events are rejected if they contain a fourth lepton with a loosened transverse momentum requirement of $p_{\mathrm {T}} > 10$ $\,\text {GeV}$, in order not to overlap with the four-lepton analysis.

These event selections have been optimized for the best precision on the expected measured cross section. A broad range of variations to the applied requirements has been considered in the optimization: including in the event selections a minimum number of jets, minimum jet $p_{\mathrm {T}}$, as well as $H_{\mathrm {T}}$; changing the number of jets required to be b-tagged; and varying the lepton momentum and isolation thresholds. Estimates of the expected backgrounds used in the optimization of the final requirements have been made both with initial estimates from simulation alone as well as with events in data control samples using the methods described below.

### Background estimation

Backgrounds passing the analysis selections are separated into three components: irreducible contributions from events with three prompt leptons and two b-quark jets (*irreducible* component), primarily with at least one top quark in the process; those with three prompt leptons and b-tagged jets without top-quark contributions (*non-top-quark* component); and contributions with at least one misidentified lepton (*misidentified lepton* component). This categorization is driven by the choice of methods used to estimate the backgrounds.

The irreducible component is split evenly among single-top-quark production in association with a ${\mathrm {Z}}$ boson (${\mathrm {t}}$ ${\mathrm {b}}$ ${\mathrm {Z}}$), ${\mathrm {t}}\overline{{\mathrm {t}}} {\mathrm {H}}$, and ${\mathrm {t}}\overline{{\mathrm {t}}} \mathrm {W}$ production; additional contributions from production of three bosons and ${\mathrm {t}}\overline{{\mathrm {t}}}$ associated with an isolated photon or two additional vector bosons are much smaller, but are still considered. Since the ${\mathrm {t}}\overline{{\mathrm {t}}} \mathrm {W}$ contribution is constrained by measurements in other (primarily the same-sign dilepton) final states, its expected SM contribution of $0.2\pm 0.1\,\text {(stat)}$ events is quoted separately. The remaining irreducible background contributions are estimated directly from simulation: $0.77\pm 0.04\,\text {(stat)} \pm 0.39\,\text {(syst)}$ events are expected. The systematic uncertainty in this background is conservatively estimated to be 50 %, dominated by the uncertainty in the cross section, in accordance with corresponding values used in Sect. 4.1. This systematic uncertainty is applied also to the ${\mathrm {t}}\overline{{\mathrm {t}}} \mathrm {W}$ contribution and serves as an initial constraint to the combined measurement, as discussed in Sect. 8.

The non-top-quark component contributions are primarily from events with three prompt leptons and b-tagged jets from misidentified light-flavour jets or b-quark jets arising from initial- or final-state radiation. In simulation, this contribution is dominated by $\mathrm {W}$ ${\mathrm {Z}}$ events. Because neither the absolute rate of extra jet production from radiation and higher-order diagrams, nor the flavour composition of additional jets are well simulated [37], we rely on data to predict this background.

A sideband sample with three leptons and no b-tagged jets, with all other selections applied, is dominated by non-top-quark backgrounds and is used to normalize the non-top-quark component prediction. The method to predict the non-top-quark backgrounds relies on the ratio $R_b$ of the number of events passing the analysis b-tagging requirements relative to those not having b-tagged jets. This ratio is assumed to be the same as for inclusive ${\mathrm {Z}}$+jets production (with the ${\mathrm {Z}}$ boson decaying leptonically) for events passing the same jet selections. We derive the $R_b$ in a sample of events with opposite-sign same-flavour leptons passing the same identification requirements as in the trilepton sample. The contribution of ${\mathrm {t}}\overline{{\mathrm {t}}}$ and other flavour-symmetric backgrounds is subtracted using opposite-flavour dilepton events after a correction for a difference in the lepton selection efficiency. For the final prediction of the non-top-quark component, an additional correction $C_b=1.4\pm 0.2\,\text {(stat)}$ is applied based on the difference between the prediction and observation in simulation. This is done to account for residual differences in the kinematic properties of jets between ${\mathrm {Z}}$+jets events and the trilepton non-top-quark background. The $R_b$ measured in dilepton events in data is $0.160\pm 0.003\,\text {(stat)}$. The non-top-quark component is predicted to contribute $2.3\pm 0.5\,\text {(stat)} \pm 1.1\,\text {(syst)}$ events. The systematic uncertainty of approximately 50 % is estimated as a combination of observed difference of $R_b$ in the dilepton events between data and simulation and the deviation of $C_b$ from unity.

Finally, the misidentified-lepton background component is estimated with a method similar to that of the same-sign dilepton analysis, described in Sect. 4.1. In each of the four final states the control sample is culled from events passing the trilepton signal event selections except that only one of the leptons is required to fail the isolation and identification requirements, still passing looser requirements. Similar to the same-sign dilepton analysis, the ratio of misidentified leptons passing full identification and isolation selections relative to the loosened requirements (the tight-to-loose ratio) is modelled to be the same in the trilepton events as in a sample with one lepton candidate and a jet. The modelling is tested in simulation, where the tight-to-loose ratio is measured in simulated multijet events and is then applied to the dominant background sample, i.e. ${\mathrm {t}}\overline{{\mathrm {t}}}$ production. The level of agreement between predicted and observed background in simulation gives the leading source of systematic uncertainty in the method, estimated to be roughly 50 %. Combined in all trilepton final states, the misidentified lepton component is estimated to be $1.2\pm 0.5\,\text {(stat)} \pm 0.6\,\text {(syst)}$ events.

### Trilepton results

**Fig. 3:**
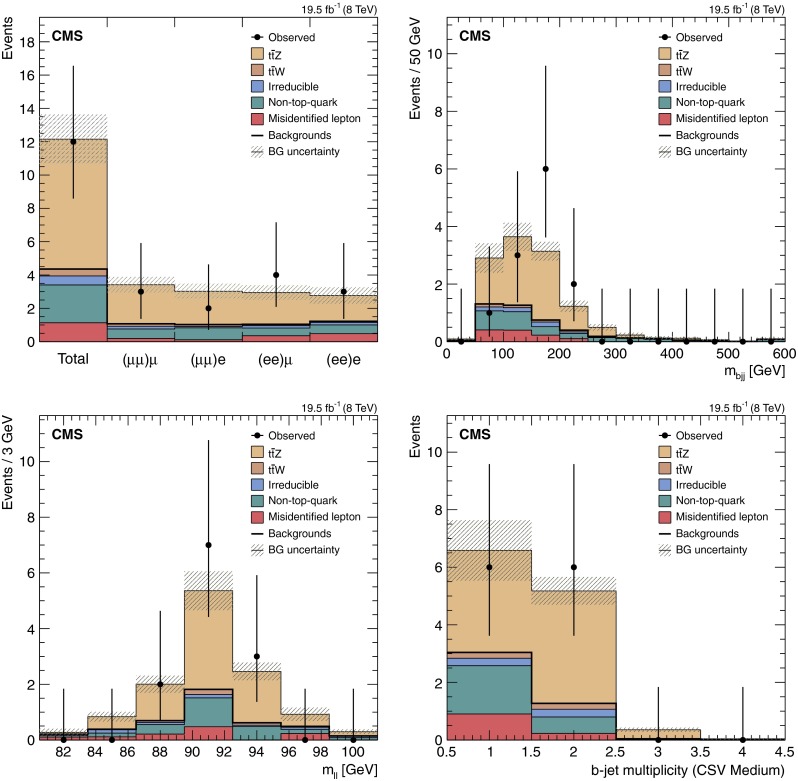
Event yields in data after final trilepton selection requirements, compared to the background estimates and signal expectations. Contributions separated by final states where the two leptons consistent with the Z boson are indicated inside parenthesis on the bin labels (*top left*), trijets mass distribution for the hadronic top-quark candidate (*top right*), ${\mathrm {Z}}$-boson candidate dilepton mass distribution (*bottom left*), and the distribution of the number of b-tagged jets passing medium operating point of the b-tagger (*bottom right*). The combination of statistical and systematic uncertainties is denoted by the *shaded area*

The 12 events observed in data are consistent with the sum of the estimated backgrounds, $4.4\pm 1.6\,(\text {syst}\oplus \text {stat})$ events, and the expected signal, $7.8\pm 0.9\,(\text {syst}\oplus \text {stat})$ events. These results are summarized in Table 2 and illustrated in Fig. 3, which shows corresponding contributions in separate channels as well as several characteristic distributions. The trijet mass for the hadronic top-quark candidate is calculated with the same method as in Sect. 4.2.

**Table 2:** Expected signal, estimated backgrounds, the sum of the two, and observed number of events for the trilepton channel. Uncertainties include both the statistical and the systematic components. The systematic uncertainty in the signal contribution does not include the theoretical uncertainty in the signal production cross section

	Yield
${\mathrm {t}}\overline{{\mathrm {t}}} {\mathrm {Z}}$ (expected)	$7.8 \pm 0.9$
Irreducible	$0.8 \pm 0.4$
${\mathrm {t}}\overline{{\mathrm {t}}} \mathrm {W}$	$0.2 \pm 0.1$
Non-top-quark	$2.3 \pm 1.2$
Misidentified lepton	$1.1 \pm 0.8$
Total background	$4.4 \pm 1.6$
Total expected	$12.2 \pm 1.8$
Observed	12

The systematic uncertainty in the cross section measurement arises from uncertainties in the background yields and in the estimate of the signal selection efficiency. For the signal event selection, the dominant sources of systematic uncertainty are the modelling of the lepton selection and the uncertainty in the jet energy scale. They produce 6 and 5 % uncertainty in the signal selection efficiency, respectively, and sum to a total of 10 % systematic uncertainty together with the other sources of uncertainty described in Sect. 7.

Based on the observed number of events, the background estimates, and the signal acceptance, of $0.0021\pm 0.0001\,\text {(stat)} \pm 0.0002\,\text {(syst)}$, the inclusive ${\mathrm {t}}\overline{{\mathrm {t}}} {\mathrm {Z}}$ production cross section in the trilepton analysis is measured as

$$\begin{aligned} \sigma _{{\mathrm {t}}\overline{{\mathrm {t}}} {\mathrm {Z}}, 3\ell } = 190 ^{+100}_{-80}\,\text {(stat)} \pm 40\,\text {(syst)} \, \text {fb}, \end{aligned}$$

including statistical and systematic uncertainties, compared to the SM expectation of $197^{+22}_{-25}\, \text {fb}$. The significance of the result over the background-only hypothesis is equivalent to 2.3 standard deviations, compared to the expected value of 2.4. This result is combined with the four-lepton analysis and the same-sign dilepton analysis, as described in Sect. 8.

## Four-lepton analysis

The aim of the four-lepton analysis is to select events originating from the process:

$$\begin{aligned} \mathrm {p}\mathrm {p}\rightarrow {\mathrm {t}}\overline{{\mathrm {t}}} {\mathrm {Z}} \rightarrow ({\mathrm {t}}\rightarrow {\mathrm {b}}\ell \nu )({\mathrm {t}}\rightarrow {\mathrm {b}}\ell \nu )({\mathrm {Z}}\rightarrow \ell \overline{\ell }). \end{aligned}$$

These events are characterized by a pair of same-flavour, opposite-sign leptons ($\mathrm {e}$ and ${\upmu }$) with an invariant mass that is close to the nominal Z-boson mass and two additional prompt leptons.

Since the branching fraction of ${\mathrm {t}}\overline{{\mathrm {t}}} {\mathrm {Z}}$ to four leptons is very low, it is a challenge to maintain high signal efficiency and at the same time reject as much background as possible. To that end, the events are separated into two categories, one of which has a significantly higher signal-to-background ratio than the other. The event selection has been optimized using the signal significance from simulated events and is summarized in the following:

1. Events must have a total of four leptons passing the lepton identification criteria described in Sect. 3. Each electron (muon) is required to have relative isolation smaller than $9\,(10)\,\%$.
2. The highest lepton $p_{\mathrm {T}}$ must be greater than 20$\,\text {GeV}$. The remaining leptons must have $p_{\mathrm {T}} > 10$ $\,\text {GeV}$.
3. Two of the leptons must form an opposite-sign same-flavour pair with the dilepton mass between 76 and 106$\,\text {GeV}$.
4. The remaining two opposite-sign leptons must not form a same-flavour pair with the dilepton mass between 76 and 106$\,\text {GeV}$.
5. At least one jet must pass the medium CSV b-tagging selection.
6. At least one other jet must pass the loose CSV b-tagging selection.

The high signal-to-background signal region requires that events pass all of the criteria above. A second signal region requires that they pass the first five conditions and fail the sixth. These two four-lepton channels are exclusive.

### Background estimation

The standard model can produce four genuine, prompt leptons through multiboson+jets production where at least two bosons decay leptonically. Backgrounds to this search include ZZ, WWZ, WZZ, ZZZ, and rarer processes. They can prove irreducible if the multiboson production is accompanied by b-tagged jets arising from the underlying event or initial-state radiation (*irreducible* background).

The contribution from irreducible background processes is estimated using MC simulations. The process with the largest contribution in the four-lepton signal regions comes from the ZZ process. The main concern with taking this background estimate solely from a simulation is how well the rate at which bottom quarks are produced is modelled. Since these bottom quarks mainly originate from initial-state radiation, this rate is estimated in a data sample of leptonically-decaying Z bosons with two additional jets. For events in this sample the probability to pass the two b-tagging criteria is found to be about 4 %. Rescaling by this number the events in the appropriate ZZ enhanced region measured in data, the background estimate is found to agree very well with the estimate from simulations. Therefore, the latter estimate is used in the analysis.

Another source of background arises when electrons and muons are incorrectly identified as prompt and isolated (*misidentified lepton* background). These can either result from misreconstruction of hadrons or from non-prompt or non-isolated leptons passing the selection criteria. Isolated tracks are used as a proxy for misidentified leptons and to calculate a “track-to-lepton” ratio, which depends on the heavy-flavour content and jet activity. The track-to-lepton ratio is determined by measuring the number of prompt, isolated tracks and the number of prompt, isolated leptons after the contribution to the leptons from electroweak processes has been subtracted. It is calculated in two control regions in data: a region with leptonic decays of Z bosons and a region with semi-leptonic decays of ${\mathrm {t}}\overline{{\mathrm {t}}}$ pairs. The two regions cover the extremes of how much heavy-flavour content is expected in different event samples. The ratio is then interpolated between these two regions using a linear mixing of the two control samples and parameterized as a function of the variable $R_\mathrm{n-p/p }$, which is the ratio of non-isolated, non-prompt tracks to non-isolated, prompt tracks in the sample. A track is defined as prompt when its transverse impact parameter is less than 200$\,\mu \text {m}$, and non-prompt otherwise. The variable $R_\mathrm{n-p/p }$ is used in the parameterization of the track-to-lepton ratio since it quantifies the amount of heavy-flavour content in the events of a given sample. The validity of the parameterization is checked in a third control region that requires one dilepton pair consistent with the Z boson and at least one b-tagged jet: for this sample, whose heavy-flavour content is expected to be in between those of the two previous control regions, $R_\mathrm{n-p/p }$ is calculated, and the predicted and observed track-to-lepton ratios are compared and found in agreement. Finally, two sideband regions with one dilepton pair consistent with the Z boson and a third lepton, and which also satisfy the two b-tagging categorizations are defined. By calculating $R_\mathrm{n-p/p }$ and using the track-to-lepton parameterization, the probability for isolated, prompt tracks to be misidentified as electrons (muons) is found equal to $7.4 \pm 2.2\,\%$ ($1.6\pm 0.5\,\%$) in these two samples. To determine the number of background events in the signal regions, the yields in the sideband regions are then multiplied by the track-to-lepton ratios and the relevant combinatoric factors depending on the number of isolated tracks present in the events. A background yield of $0.1 \pm 0.1$ ($0.5 \pm 0.2$) in the 2 b-jet (1 b-jet) signal region is calculated in this way.

### Four-lepton results

**Fig. 4:**
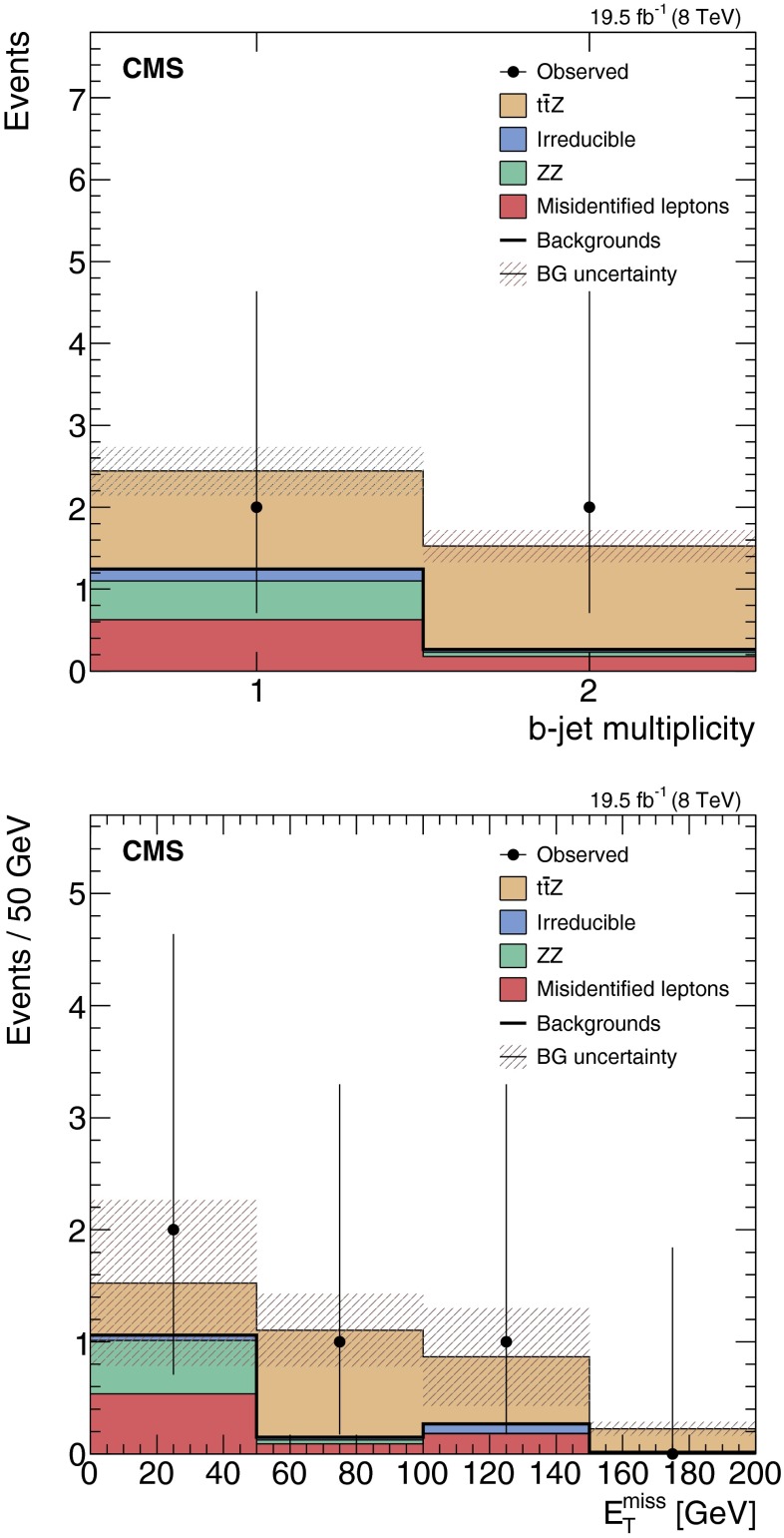
Event yields in data after final four-lepton selection requirements, compared to the background estimates and signal expectations. Contributions in the 1 b-tagged jet and 2 b-tagged jets signal regions (*top*) and inclusive $E_{\mathrm {T}}^{\text {miss}}$ distribution (*bottom*). The ZZ component of the background is shown separately from the rest of the irreducible processes. The combination of statistical and systematic uncertainties is denoted by the *shaded area*

Applying the full event selection, the event yields shown in Table 3 are obtained. A total of 4 events are observed, compared to a background expectation of $1.4 \pm 0.3$ events, where the uncertainty in the background prediction contains both the contributions from the limited number of simulated events and from the uncertainties related to the rescaling procedure based on control samples in data. The results are shown in Fig. 4 (top). A comparison of the $E_{\mathrm {T}}^{\text {miss}}$ distributions for the background, signal, and observed data, combining the two signal regions, is shown in Fig. 4 (bottom).

**Table 3:** Expected signal, estimated backgrounds, the sum of the two, and observed number of events for the four-lepton channel. Uncertainties include both the statistical and the systematic components. The systematic uncertainty in the signal contribution does not include the theoretical uncertainty in the signal production cross section. The ZZ component of the background is shown separately from the rest of the irreducible processes

	2 b Jets required	1 b Jet required
${\mathrm {t}}\overline{{\mathrm {t}}} {\mathrm {Z}}$ (expected)	1.3 $\pm$ 0.2	1.3 $\pm$ 0.2
Misidentified lepton	0.1 $\pm$ 0.1	0.5 $\pm$ 0.2
ZZ	0.05 $\pm$ 0.01	0.47 $\pm$ 0.02
Irreducible	0.04 $\pm$ 0.03	0.14 $\pm$ 0.04
Total background	0.2 $\pm$ 0.1	1.1 $\pm$ 0.2
Total expected	1.5 $\pm$ 0.2	2.4 $\pm$ 0.3
Observed	2	2

The systematic uncertainties in the selection efficiencies for signal and irreducible background are derived in the same way as for the dilepton and trilepton channels and are described in Sect. 7. For the four-lepton analysis, the dominant source of uncertainty in the signal acceptance is the 8 % uncertainty in the modelling of the lepton selection. Together with the other systematic uncertainties, it sums to a total uncertainty of 11 % in the signal selection efficiency.

By performing a simultaneous fit to the two exclusive four-lepton signal regions, the following cross section is extracted:

$$\begin{aligned} \sigma _{{\mathrm {t}}\overline{{\mathrm {t}}} {\mathrm {Z}}, 4\ell } = 230 ^{+180}_{-130}\,\text {(stat)} ^{+60}_{-30}\,\text {(syst)} \, \text {fb}. \end{aligned}$$

The significance is equal to 2.2 standard deviations (2.0 standard deviations expected).

## Systematic uncertainties in signal selection efficiency

Along with the corresponding techniques for the background estimation, the uncertainties in the estimates of the backgrounds affecting the three channels have been presented respectively in Sects. 4, 5, and 6. Here are illustrated the uncertainties in the selection efficiency of signal events.

Except for the component due to trigger, simulation is used to estimate the selection efficiency for signal. Control samples in data are used to correct the selection acceptance estimated in simulation and to assess the corresponding uncertainty. A similar approach is employed for all three analysis channels and therefore a common list of systematic uncertainties in signal acceptance can be summarized as in Table 4. The total uncertainty in the computed acceptance varies between 8 and 11 % depending on the channel.

**Table 4:** Systematic uncertainties in the signal selection efficiency for the three considered channels: ${\mathrm {t}}\overline{{\mathrm {t}}} \mathrm {W}$ in dilepton (2$\ell$) final state; ${\mathrm {t}}\overline{{\mathrm {t}}} {\mathrm {Z}}$ in trilepton (3$\ell$) and four-lepton (4$\ell$) final states

Source of uncertainty	Channels		
	2$\ell$	3$\ell$	4$\ell$
	Uncertainty (%)		
Modelling of trigger eff.	3	1	1
Modelling of lepton sel. (ID/isolation)	4	6	8
Jet energy scale and resolution	4	5	4
Identification of b jets	2	3	3
Pileup modelling	1	1	1
Choice of parton distribution functions	1.5	1.5	1.5
Signal model	5	5	5
Total	8	10	11

The trigger efficiency is directly measured in data using control samples selected by $H_{\mathrm {T}}$ triggers that are orthogonal to the dilepton triggers employed by the three analyses to select signal event candidates [9]. Trigger inefficiencies are then applied to all acceptances calculated from simulation, for both signal and the background processes derived from simulation.

The offline lepton selection efficiencies in data and simulation are measured using ${\mathrm {Z}}$-boson events to derive simulation-to-data correction factors. The correction factors applied to simulation are about 0.94 (0.98) for $p_{\mathrm {T}}$ $>$ 20$\,\text {GeV}$ for electrons (muons). The uncertainty in the per-lepton selection efficiency is about 1.5 % (0.3 %) for electrons (muons) with $p_{\mathrm {T}}$ $> 20$ $\,\text {GeV}$. An additional systematic uncertainty is assigned to account for potential mismodelling of the lepton isolation efficiency due to the larger hadronic activity in signal events than in ${\mathrm {Z}}$-boson events. This uncertainty is in the 2–3 % range. These per-lepton uncertainties are propagated to calculate the uncertainties in the selection efficiency of signal events, which are found to be in the 4–8 % range depending on the leptonic final state.

Another source of systematic uncertainty is associated with the jet energy scale correction. This systematic uncertainty varies between 5 and 2 % in the $p_{\mathrm {T}}$ range 40–100$\,\text {GeV}$ for jets with $|\eta |<2.4$ [22]. It is evaluated on a single-jet basis, and its effect is propagated to $H_{\mathrm {T}}$, the number of jets, and the number of b-tagged jets. In addition, there is a contribution to the total uncertainty arising from limited knowledge of the resolution of the jet energy, but this effect is generally of less importance than the contribution from the jet energy scale.

The b-tagging efficiency for b-quark jets, and the mistagging probabilities for charm-quark jets and for jets originating from light-flavour quarks or gluons, are estimated from data [38]. The corresponding correction factors, dependent on jet flavour and kinematic properties, are applied to simulated jets to account for the differences in the tagging efficiency between simulation and data. The total uncertainty in the signal acceptance caused by the b-tagging selection is determined by varying the correction factors up and down by their uncertainties.

In the simulation of signal events, different pileup conditions have been probed varying the cross section for inelastic pp collisions by $\pm$5 %. Comparing the signal selection efficiency for these different conditions, the uncertainty associated to pileup effects is found to be approximately 1 %. The uncertainty in the signal acceptance due to the PDF choice [13, 39–42] is found to be 1.5 %. An uncertainty of the order of 5 % in the signal acceptance is also assigned to the finite-order calculation employed to generate signal events. This last uncertainty, which covers also the uncertainty in the effects of initial- and final-state radiation, is estimated varying from their nominal values the matrix-element/parton-shower matching scale (with the nominal value of 20$\,\text {GeV}$), and the renormalization and factorization scales (with the nominal value equal to $Q^2$ in the event). For the up and down variations of the matching scale, thresholds of 40 and 10$\,\text {GeV}$ are used, respectively. Renormalization and factorization scales are varied between 4Q$^2$ and Q$^2$/4. The signal model uncertainty also includes the difference in acceptance between signal events simulated with MadGraph 5 and amc@nlo  [15] generators.

## Results

To extract the cross sections for the ${\mathrm {t}}\overline{{\mathrm {t}}} \mathrm {W}$ and ${\mathrm {t}}\overline{{\mathrm {t}}} {\mathrm {Z}}$  processes, the nine different channels are combined to maximize their sensitivity. Cross section central values and corresponding uncertainties are evaluated from a scan of the profile likelihood ratio. The adopted statistical procedure is the same that was used for the observation of the Higgs boson candidate in CMS, and is described in detail in Ref. [29].

The results of the measurements are summarized in Table 5. Two one-dimensional fits are performed to measure ${\mathrm {t}}\overline{{\mathrm {t}}} \mathrm {W}$ and ${\mathrm {t}}\overline{{\mathrm {t}}} {\mathrm {Z}}$ separately using the channels most sensitive to each process. Using only the same-sign dilepton channels, the extracted ${\mathrm {t}}\overline{{\mathrm {t}}} \mathrm {W}$ cross section is measured to be $170 ^{+90}_{-80}\,\text {(stat)} \pm 70\,\text {(syst)}$ $\, \text {fb}$, corresponding to a significance of $1.6$ standard deviations over the background-only hypothesis. The three and four lepton channels are combined to extract a ${\mathrm {t}}\overline{{\mathrm {t}}} {\mathrm {Z}}$ cross section of $200 ^{+80}_{-70}\,\text {(stat)} ^{+40}_{-30}\,\text {(syst)}$ $\, \text {fb}$, with a significance of 3.1 standard deviations.

**Table 5:** Results of the extraction of cross sections, from single and combined channels. The significance is expressed in terms of standard deviations

Channels used	Process	Cross section	Significance
2$\ell$	${\mathrm {t}}\overline{{\mathrm {t}}} \mathrm {W}$	$170 ^{+90}_{-80}\,\text {(stat)} \pm 70\,\text {(syst)}$ $\, \text {fb}$	1.6
3$\ell$+4$\ell$	${\mathrm {t}}\overline{{\mathrm {t}}} {\mathrm {Z}}$	$200 ^{+80}_{-70}\,\text {(stat)} ^{+40}_{-30}\,\text {(syst)}$ $\, \text {fb}$	3.1
2$\ell$+3$\ell$+4$\ell$	${\mathrm {t}}\overline{{\mathrm {t}}} \mathrm {W}$ $+$ ${\mathrm {t}}\overline{{\mathrm {t}}} {\mathrm {Z}}$	$380 ^{+100}_{-90}\,\text {(stat)} ^{+80}_{-70}\,\text {(syst)}$ $\, \text {fb}$	3.7

When calculating the one-dimensional fit of one process, the cross section of the other process is constrained to have the theoretical SM value with a systematic uncertainty of 50 %.

As visible from Fig. 2 and Table 1, less than 10 % of the events selected by the same-sign dilepton channels are expected to stem from ${\mathrm {t}}\overline{{\mathrm {t}}} {\mathrm {Z}}$ production. The extracted ${\mathrm {t}}\overline{{\mathrm {t}}} \mathrm {W}$ cross section varies by approximately 10 % when the used ${\mathrm {t}}\overline{{\mathrm {t}}} {\mathrm {Z}}$ cross section is altered to as much as 0.5–1.5 times its nominal theoretical value. For an equivalent modification of the ${\mathrm {t}}\overline{{\mathrm {t}}} \mathrm {W}$ production rate, the variation of the extracted ${\mathrm {t}}\overline{{\mathrm {t}}} {\mathrm {Z}}$ cross section is less than 2 %. The dependence of the measured cross section on the assumed cross section of the other ${\mathrm {t}}\overline{{\mathrm {t}}} {\mathrm {V}}$ process is solved by performing a simultaneous fit of the cross sections of the two processes using all dilepton, trilepton, and four-lepton channels at the same time.

**Fig. 5:**
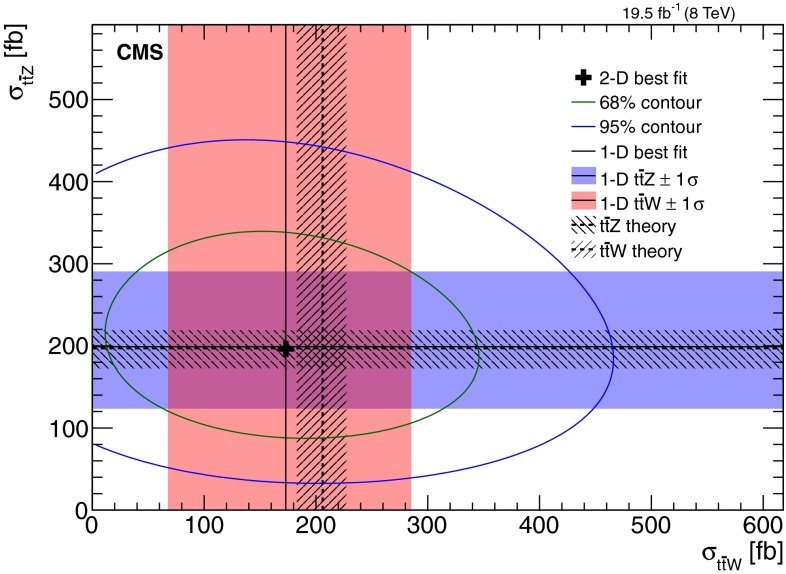
The result of the two-dimensional best fit for ${\mathrm {t}}\overline{{\mathrm {t}}} \mathrm {W}$ and ${\mathrm {t}}\overline{{\mathrm {t}}} {\mathrm {Z}}$ cross sections (*cross symbol*) is shown along with its 68 and 95 % confidence level contours. The result of this fit is superimposed with the separate ${\mathrm {t}}\overline{{\mathrm {t}}} \mathrm {W}$ and ${\mathrm {t}}\overline{{\mathrm {t}}} {\mathrm {Z}}$ cross section measurements, and the corresponding 1 standard deviation (1$\sigma$) bands, obtained from the dilepton, and the trilepton/four-lepton channels, respectively. The figure also shows the predictions from theory and the corresponding uncertainties

The result of the fit is shown visually in Fig. 5 and the cross sections are summarized numerically in Table 6. The cross sections extracted from this two-dimensional fit are identical to those obtained from the two one-dimensional fits.

**Table 6:** Results for the two dimensional fit of the ${\mathrm {t}}\overline{{\mathrm {t}}} \mathrm {W}$ and ${\mathrm {t}}\overline{{\mathrm {t}}} {\mathrm {Z}}$ cross sections

Channels used	${\mathrm {t}}\overline{{\mathrm {t}}} \mathrm {W}$ cross section	${\mathrm {t}}\overline{{\mathrm {t}}} {\mathrm {Z}}$ cross section
2$\ell$+3$\ell$+4$\ell$	$170 ^{+110}_{-100}\,\text {(total)}$ $\, \text {fb}$	$200 \pm 90\,\text {(total)}$ $\, \text {fb}$

Finally, a one-dimensional fit of all channels is performed to extract a combined cross section $\sigma _{{\mathrm {t}}\overline{{\mathrm {t}}} {\mathrm {V}} } = 380 ^{+100}_{-90}\,\text {(stat)} ^{+80}_{-70}\,\text {(syst)}$ $\, \text {fb}$ with a significance of 3.7 standard deviations.

## Summary

A measurement with the CMS detector of the cross section of top quark–antiquark pair production in association with a W or Z boson at $\sqrt{s} = 8$ $\,\text {TeV}$ has been presented. Results from three independent channels, and their combination, have been reported. In the same-sign dilepton channel, the ${\mathrm {t}}\overline{{\mathrm {t}}} \mathrm {W}$ cross section has been measured to be $\sigma _{{\mathrm {t}}\overline{{\mathrm {t}}} \mathrm {W}} = 170 ^{+90}_{-80}\,\text {(stat)} \pm 70\,\text {(syst)} \, \text {fb}$, corresponding to a significance of 1.6 standard deviations over the background-only hypothesis. In the trilepton and four-lepton channels the ${\mathrm {t}}\overline{{\mathrm {t}}} {\mathrm {Z}}$ signal has been established with a significance of 2.3 and 2.2 standard deviations, respectively. From the combination of these two channels, a significance of 3.1 standard deviations has been obtained and the cross section has been measured to be $\sigma _{{\mathrm {t}}\overline{{\mathrm {t}}} {\mathrm {Z}}} = 200 ^{+80}_{-70}\,\text {(stat)} ^{+40}_{-30}\,\text {(syst)} \, \text {fb}$.

Combining the total of nine sub-channels from the three lepton decay modes, a ${\mathrm {t}}\overline{{\mathrm {t}}} {\mathrm {V}}$ cross section (V equal W or Z) of $\sigma _{{\mathrm {t}}\overline{{\mathrm {t}}} {\mathrm {V}} } = 380 ^{+100}_{-90}\,\text {(stat)} ^{+80}_{-70}\,\text {(syst)} \, \text {fb}$ has been obtained, corresponding to a combined significance of 3.7 standard deviations. The measured values are compatible within their uncertainties with standard model predictions.

## References

[1] CDF Collaboration, Observation of top quark production in $$\overline{\mathit{p}}\mathit{p}$$ collisions with the collider detector at fermilab. Phys. Rev. Lett. **74**, 2626 (1995). doi:10.1103/PhysRevLett.74.2626. arXiv:hep-ex/9503002 10.1103/PhysRevLett.74.2626 10057978

[2] D0 Collaboration, Observation of the top quark. Phys. Rev. Lett. **74**, 2632 (1995). doi:10.1103/PhysRevLett.74.2632 10.1103/PhysRevLett.74.2632 10057979

[3] Glashow SL (1961). Partial-symmetries of weak interactions. Nucl. Phys..

[4] S. Weinberg, A model of leptons. Phys. Rev. Lett. **19**, 1264 (1967). doi:10.1103/PhysRevLett.19.1264

[5] A. Salam, in *Elementary Particle Physics: Relativistic Groups and Analyticity*. Weak and Electromagnetic Interactions. Proceedings of the Eighth Nobel Symposium, ed. by N. Svartholm (Almqvist & Wiskell, 1968), p. 367

[6] Barnett RM, Gunion JF, Haber HE (1993). Discovering supersymmetry with like-sign dileptons. Phys. Lett. B.

[7] Guchait M, Roy DP (1995). Like-sign dilepton signature for gluino production at CERN LHC including top quark and Higgs boson effects. Phys. Rev. D.

[8] Baer H, Chen C-H, Paige F, Tata X (1996). Signals for minimal supergravity at the CERN large hadron collider. II: Multi-lepton channels. Phys. Rev. D.

[9] CMS Collaboration, Search for new physics in events with same-sign dileptons and jets in pp collisions at $$\sqrt{s}$$ = 8 TeV. JHEP **01**, 163 (2014). doi:10.1007/JHEP01(2014)163. arXiv:1311.6736

[10] CMS Collaboration, Search for anomalous production of multilepton events in pp collisions at $$\sqrt{s}=7$$ TeV. JHEP **06**, 169 (2012). doi:10.1007/JHEP06(2012)169. arXiv:1204.5341

[11] CDF Collaboration, Evidence for $$t\overline{t}\gamma $$ Production and Measurement of $$\sigma _{{\rm t}\mathit{\bar{{\rm t}}}\gamma } / \sigma _{{\rm t}\mathit{\bar{{\rm t}}}}$$. Phys. Rev. D **84**, 031104 (2011). doi:10.1103/PhysRevD.84.031104. arXiv:1106.3970

[12] CMS Collaboration, Measurement of associated production of vector bosons and top quark-antiquark pairs at $$\sqrt{s}$$ = 7 TeV. Phys. Rev. Lett. **110**, 172002 (2013). doi:10.1103/PhysRevLett.110.172002. arXiv:1303.3239 10.1103/PhysRevLett.110.172002 23679709

[13] Lai H-L (2010). New parton distributions for collider physics. Phys. Rev. D.

[14] J. Alwall et al., The automated computation of tree-level and next-to-leading order differential cross sections, and their matching to parton shower simulations. JHEP **07**, 079 (2014). doi:10.1007/JHEP07(2014)079. arXiv:1405.0301

[15] Frixione S, Webber BR (2002). Matching NLO QCD computations and parton shower simulations. JHEP.

[16] J.M. Campbell, R.K. Ellis, $$t\overline{t}W^\pm $$ production and decay at NLO. JHEP **07**, 052 (2012). doi:10.1007/JHEP07(2012)052. arXiv:1204.5678

[17] M.V. Garzelli, A. Kardos, C.G. Papadopoulos, Z. Trocsanyi, $${\rm t}{\bar{{\rm t}}}{\rm W}^{\pm }$$ and $${\rm t}\bar{{\rm t}{\rm Z}}$$ hadroproduction at NLO accuracy in QCD with Parton Shower and Hadronization effects. JHEP **11**, 056 (2012). doi:10.1007/JHEP11(2012)056. arXiv:1208.2665

[18] CMS Collaboration, The CMS experiment at the CERN LHC. JINST **3**, S08004 (2008). doi:10.1088/1748-0221/3/08/S08004

[19] CMS Collaboration, Particle-Flow Event Reconstruction in CMS and Performance for Jets, Taus, and $$E_{T}^{{\rm miss}}$$”, CMS Physics Analysis Summary. CMS-PAS-PFT-09-001 (2009)

[20] CMS Collaboration, Commissioning of the Particle-flow Event Reconstruction with the first LHC collisions recorded in the CMS detector, CMS Physics Analysis Summary. CMS-PAS-PFT-10-001 (2010)

[21] Cacciari M, Salam GP, Soyez G (2008). The anti-$$k_t$$ jet clustering algorithm. JHEP.

[22] CMS Collaboration, Determination of jet energy calibration and transverse momentum resolution in CMS. JINST **6**, P11002 (2011). doi:10.1088/1748-0221/6/11/P11002. arXiv:1107.4277

[23] Cacciari M, Salam GP, Soyez G (2008). The catchment area of jets. JHEP.

[24] CMS Collaboration, Measurements of differential jet cross sections in proton-proton collisions at $$\sqrt{s}=7$$ TeV with the CMS detector. Phys. Rev. D **87**, 112002 (2013). doi:10.1103/PhysRevD.87.112002. arXiv:1212.6660

[25] CMS Collaboration, Pileup Jet Identification, CMS Physics Analysis Summary. CMS-PAS-JME-13-005 (2013)

[26] CMS Collaboration, Performance of b tagging at $$\sqrt{s}$$=8 TeV in multijet, ttbar and boosted topology events, CMS Physics Analysis Summary. CMS-PAS-BTV-13-001 (2013)

[27] CMS Collaboration, Performance of CMS muon reconstruction in pp collision events at $$\sqrt{s}=7$$ TeV. JINST **7**, P10002 (2012). doi:10.1088/1748-0221/7/10/P10002. arXiv:1206.4071

[28] CMS Collaboration, Electron Reconstruction and Identification at $$\sqrt{s} = 7$$ TeV, CMS Physics Analysis Summary. CMS-PAS-EGM-10-004 (2010)

[29] CMS Collaboration, Observation of a new boson with mass near 125 GeV in pp collisions at $$\sqrt{s}$$ = 7 and 8 TeV. JHEP **06**, 081 (2013). doi:10.1007/JHEP06(2013)081. arXiv:1303.4571

[30] J. Alwall et al., MadGraph 5: going beyond. JHEP **06**, 128 (2011). doi:10.1007/JHEP06(2011)128. arXiv:1106.0522

[31] T. Sjöstrand, S. Mrenna, P.Z. Skands, Pythia 6.4 physics and manual, JHEP **05** 026 (2006). doi: 10.1088/1126-6708/2006/05/026. arXiv:hep-ph/0603175

[32] GEANT4 Collaboration, Geant4—a simulation toolkit. Nucl. Instrum. Meth. A **506**, 250 (2003). doi:10.1016/S0168-9002(03)01368-8

[33] Nadolsky PM (2008). Implications of CTEQ global analysis for collider observables. Phys. Rev. D.

[34] Particle Data Group Collaboration, Phys. Rev. D Review of particle physics. **86**, 010001 (2012). doi:10.1103/PhysRevD.86.010001

[35] CMS Collaboration, Study of double parton scattering using W + 2-jet events in proton-proton collisions at $$\sqrt{s}$$ = 7 TeV. JHEP **03**, 032 (2014). doi:10.1007/JHEP03(2014)032. arXiv:1312.5729

[36] CMS Collaboration, CMS Luminosity Based on Pixel Cluster Counting-Summer 2013 Update, CMS Physics Analysis Summary. CMS-PAS-LUM-13-001 (2013)

[37] CMS Collaboration, Measurement of $$B\bar{B}$$ angular correlations based on secondary vertex reconstruction at $$\sqrt{s}=7$$ TeV. JHEP **03**, 136 (2011). doi:10.1007/JHEP03(2011)136. arXiv:1102.3194

[38] CMS Collaboration, Identification of b-quark jets with the CMS experiment. JINST **8**, P04013 (2013). doi:10.1088/1748-0221/8/04/P04013. arXiv:1211.4462

[39] S. Alekhin et al., The PDF4LHC Working Group Interim Report (2011) arXiv:1101.0536

[40] M. Botje et al., The PDF4LHC Working Group Interim Recommendations (2011). arXiv:1101.0538

[41] NNPDF Collaboration, Impact of heavy quark masses on parton distributions and LHC Phenomenology, Nucl. Phys. B **849**, 296 (2011). doi: 10.1016/j.nuclphysb.2011.03.021. arXiv:1101.1300

[42] Martin AD, Stirling WJ, Thorne RS, Watt G (2009). Parton distributions for the LHC. Eur. Phys. J. C.

